# A study of different cognitive states for meditators and non-meditators with the use of multiple classification indices derived from the PSD of EEG data and lessons learned about cognitive states and the nature of intelligence in minds and machines

**DOI:** 10.3389/fnsys.2025.1718733

**Published:** 2026-01-23

**Authors:** J. J. Joshua Davis, Florian Schübeler, Ian J. Kirk, Robert Kozma

**Affiliations:** 1 The Embassy of Peace, Whitianga, Coromandel, New Zealand; 2 MINDLab, School of Psychology and Centre for Brain Research, University of Auckland, Auckland, New Zealand; 3 Department of Mathematics, University of Memphis, Memphis, TN, United States; 4 School of Informatics, Obuda University, Budapest, Hungary; 5 Kozma Research Lab, Boston, MA, United States

**Keywords:** awareness, cognition, EEG, intentionality, meaning, meditation, Shannon Entropy, skewness

## Abstract

This study explores the layered coherence within human cognition as measured through EEG. Signals were collected from two groups (meditators vs. non-meditators) across six conditions: Meditation, Scrambled Words, Ambiguous Images, Math Mind, Sentences, and Video Watching. We analyzed the EEG data using Shannon Entropy, Pearson’s Skewness, Total Power, and Dominant Frequency indices, now taken together, to reveal distinct neurophysiological signatures and a different outcome of hypothesis testing based on one index at a time only. These patterns suggest that cognition is more than merely computational, since it seems to be expressive of deeper experiential states, raising profound questions about the nature of intelligence and whether the human psyche and its experience of meaning, in its different forms, can be meaningfully approached through objective methodologies. Our findings invite a re-examination of scientific inquiry itself, both as a pursuit of mechanistic regularities, and also, holistically, as a means of honoring the subtle interplay between structure and meaning. This is reminiscent of young Carl Friedrich Gauss revealing hidden structure beneath apparent complexity by summing up an arithmetic series with elegant simplicity. This way he reframed a problem through insight rather than brute calculation. If artificial intelligence is to mimic cognition, it must grapple with informational entropy and also with the values and consciousness that give rise to meaning. The entropic balance of EEG signals may offer a window into coherence, yet only a species that is mature enough to honor life, liberty, and the pursuit of deep meaning, should attempt to design artificial “minds.” In this convergence of neuroscience and philosophical reflection, we glimpse a deeper imperative: to preserve the truth of what it means to be human in an age increasingly defined by machines.

## 1 Introduction

Signals coming from the environment, the ecological and ethological niches of an individual, via visual, auditory and kinesthetic stimuli, together with self-regulating activities like rhythmic breathing, relaxation and meditative practices, all contribute to the behavior of different oscillatory systems in the body, like the heart, the brain, the respiratory system, and the digestive system among others.

The selection of five engaged tasks alongside a single meditative/relaxed condition reflects the central aim of the study: to contrast meditative and relaxed states, characterized in previous studies by Alpha dominance and reduced cognitive demand, with a range of sensory and cognitively active modalities known to elicit broader spectral engagement across Theta, Alpha, Beta, and Gamma bands. This design enabled us to examine how differing levels of multi-sensory environmental stimulation and task-related processing shape brain dynamics within a unified methodological framework. The intent was to characterize systematic differences across modalities using entropy-, skewness-, and frequency-based indices, rather than to support or construct a theory of consciousness. The broader reflections on cognition and intelligence that appear later in the manuscript arise only after the empirical distinctions between modalities were established and serve as conceptual commentary, separate from the experimental rationale, which is grounded in established systems-neuroscience findings on oscillatory activity under presumably varying cognitive demands.

In order to understand the different kinds of brain dynamics caused by the combination of environmental stimuli and psychophysiological self-regulation, in recent years, we developed a methodology that invokes diverse mathematical modeling techniques, systems theory, systems dynamics, dynamical systems and chaos∼order theory (Davis et al., 2025). We have applied such methodology when studying human intentionality and decision-making grounded in meaning and knowledge creation, to evaluate different cognitive states, including meditative states, for example, inspired by the work of Austin (1999), Buzsáki (2006), Davis (2009), Freeman et al. (2000), Kasamatsu and Hirai (1966), Kozma et al. (2012), and Schwartz et al. (2005).

Previous studies have shown that sensory input causes neural activity in various frequency bands (Freeman et al., 2003; Freeman and Quiroga, 2013; Kozma and Freeman, 2016). Other authors have shown that such frequency spectrum manifests in experiments when individuals are exposed to auditory input from verbal stimuli, performance of arithmetic operations via verbal request and meditation practices, for example (Austin, 1999; Davis et al., 2023b,2024b; Kasamatsu and Hirai, 1966). We have made good progress through the years in formalizing and applying our methodology (Davis et al., 2025; Davis et al., 2015b; Davis et al., 2016; Davis et al., 2017; Davis et al., 2023b, 2023a, 2024b; 2024a), and have gained more insight about different brain states in different engaged modalities^1^ presumably associated to more energy demanding states in contrast to meditative states. Here, meditation and relaxation will serve as a baseline for contrasting different cognitive states. It is important to note that activity over the Alpha band is observed during meditative states associated with reports of inner peace (Austin, 1999; Buzsáki, 2006; Davis, 2009; Echenhofer and Coombs, 1987; Freeman et al., 2000; Kasamatsu and Hirai, 1966; Schwartz et al., 2005). This means that Alpha dominance could be used as a marker or baseline when measuring different cognitive states that may contrast with meditative states, and that show activity in Gamma or Beta for example, as the dominant bands activity in diverse regions of the brain, when performing more demanding tasks.

We use our methodologies as described in Davis et al. (2025), Davis and Kozma (2018), Davis et al. (2016), Davis et al. (2019b), and Davis et al. (2020), by applying a statistical evaluation of new experimental EEG data, obtained in Ian J. Kirk’s Lab, Centre for Brain Research at The University of Auckland in New Zealand, during a period of around 3 months of data acquisition in six modalities, for 20 participants.

We use the Shannon Entropy index (H), Pearson’s 1st skewness coefficient (PSk), Total Power (TP) and Dominant Frequency based indices (DF and DFs) to discrimination between different brain states. We perform an extended analysis of the EEG array data in these six modalities that show that all the H, PSk, TP and DF based indices are able to significantly discriminate between brain dynamics in different modalities, presumably associated to different brain, psychophysiological and inner states.

We perform a pairwise comparison to test a set of hypotheses based on equality of means (for each index) between modalities. This allows us to statistically infer differences between modalities and groups, and in so doing, draw some conclusions about the behavior of the different cognitive states associated with each modality, as represented by different brain behavior based on H, PSk, TP, DFs.

We observe a contrasting difference between participants and between modalities. We study whether busy states require more information processing and energy consumption, and whether they are more entropic, or less ordered, than meditative states. We also study the relative difference in entropy between the busier states. It appears that in general, meditative or relaxing states should differ significantly from more busy states. We aim at showing that the indices derived from brain dynamics measured in the more engaged modalities, may be associated to a more diverse kind of Power Spectrum, manifesting a broader range of frequencies, like the Theta, Alpha, Beta and Gamma bands in different brain areas (Freeman et al., 2003; Freeman and Quiroga, 2013), and significantly contrasting with the Power Spectrum associated to measured brain activity in meditative or relaxed states, where the Alpha band is expected to be dominant for certain brain regions.

Next, we discuss the Artificial Intelligence paradigm. As a consequence of the introduced results and drawing upon our accumulated experience and knowledge of human intelligence, the discussion section explores the differences we find most salient between Human and Artificial “Intelligence.” Since the differences are closely related to human consciousness and cognition in meditative states, in the conclusion section, we briefly turn to Baar’s take on silent consciousness (Baars, 2013). We conclude this work by outlining avenues for future studies.

## 2 Description of the experiment

The EEG technology used for this study was a HydroCel Geodesic Sensor Net (HCGSN), 128 electrodes dense-array electroencephalography (EEG) produced by Electrical Geodesics, Inc. (Electrical Geodesics Inc, 2007). We also used E-Prime 2.0 to program the experimental setting and the presentation of stimuli, as well as relevant events like “start” and “press key.”

**FIGURE 1:**
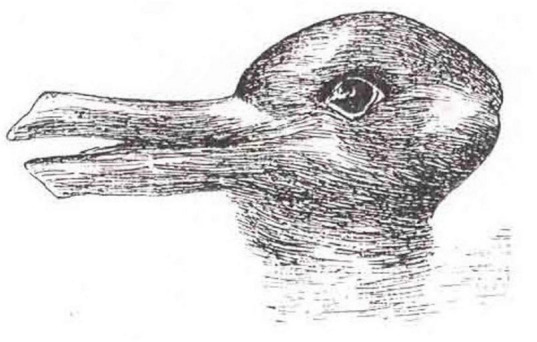
Duck-rabbit optical illusion illustrating perceptual ambiguity. Depending on viewer interpretation, the image can be seen as either a duck facing left or a rabbit facing right. Originally created by Jastrow (1899), adapted and published under Creative Commons Attribution-Share Alike 3.0 via Wikimedia Commons.

We expand on earlier studies (Davis and Kozma, 2018; Davis et al., 2023b; Davis et al., 2020), to include 20 participants that were measured in six modalities, as follows:

- Meditation (MED): Participants were asked to engage in the meditation of their choice for 7 min with their eyes closed. Alternatively, if people were unfamiliar with meditating, they were asked to relax with their eyes closed.
- Scrambled Words (WORDS): Participants were presented 20 scrambled words that pertained to one of the two categories, either a value, such as Love or Truth, for example, or an object from nature, such as Sand or Pebble, for example. The participants were asked to mentally unscramble the word and once they had established the category of the word, they were asked to press number 1 on the number pad when they identified the word describing a “Value” or to press number 2 on the number pad when the word was describing an “Object.”
- Ambiguous Images (IMG): We presented 12 images with ambiguous content to the participants and asked them to specify how many individual images they could find by pressing a number between 1 and 9 on the number pad, in order to identify the number of images found. See Figure 1 with two different images in it, for an example.
- Math Mind (MM): For this modality we recorded a pleasant female voice presenting 28 simple arithmetic operations that the participants were asked to resolve in their mind by applying some rules, until they reached a solution with a single digit between one and nine, for each arithmetic operation. For example, the participant would hear “7 × 7,” which equates to 49, a two-digit result. Then the participant would add these two digits together (i.e., 4+9 = 13) and repeat this step until they reached a single digit result. In this case the answer would be “4,” after the final computation of “1+3” was performed.
- Sentences (SENT): Twenty sentences were recorded with the same female voice as for the MM modality. The sentence was comprised of either a positive statement, such as: “You are caring and kind” or a gibberish sentence, such as “briggy tublish tuchty.” The participants were asked to categorize the sentence they heard, as either meaningless, meaningful or meaningful and pleasant, and to press the corresponding number 1, 2, or 3 on the number pad.
- Video (VDO): In this final modality, the participants were presented with a video of ambiguous images and the song “Imagine” by John Lennon playing throughout the video. There was no task the participants were asked to perform, besides watching the video.

For the experiment, the participant was inside a Faraday chamber, positioned in a comfortable chair facing a computer screen with a standard computer keyboard in front of them. Participants were instructed to use only the space bar and the number pad, and to restrict head and eye movement as much as possible to minimize artifacts in the EEG data recording. An impedance check was done prior to the beginning of the experiment. There were three experimental blocks, with MED and WORDS in block 1, followed by a break where the impedance of the EEG electrodes was measured again and adjusted when needed. Then block 2 was started, comprised of IMG and MM, followed by the same impedance calibration procedure as before. The experiment concluded with block 3, SENT and VDO.

The 20 participants (P1–P20), were all in good health, comprised of 11 males and nine females, within an age range from 23 to 64 years. Some practice meditation regularly and others have none or little experience in meditation practices. We assigned 11 participants to the *Meditator* group, those who have been regularly practicing meditation for at least 2 years and for a minimum of 5 days a week. The remaining nine were assigned to the *Non-Meditator* group.

All participants, ranging from 2) to 64 years of age, were healthy and cognitively sound, as they included student volunteers, professors, and individuals actively engaged in professional work. The given age range was never by design, rather it was due to the limitations of the available participants. Future experiments need to explore a broader age range of population. Participants were required to be unmedicated and free from the influence of substances such as drugs, alcohol, or caffeine for at least 24 h prior to the experiment. Formal cognitive or medical screening was never conducted in this pilot study to fully guarantee general and mental and cognitive health; such screening may be considered in future investigations, when required, to further ensure homogeneity and control for potential confounds.

## 3 Methods and signal processing

We designed the experiment with careful attention to task order and the potential transfer effects between modalities. We felt that starting with meditation and relaxation would set an initial positive tone for the participant. The transfer effects of this initial positive tone is outside the scope of this study, especially given the small sample size of 20 participants and its pilot nature and the limitations that the number of participants imposes, given it is a highly sensitive parameter in an experimental study of carryover effects between cognitive states. The goal was to obtain preliminary indications of how meditative or relaxed states differ from more cognitively engaged states. The session began with Block 1, 7 min of meditation/relaxation to ease participants into the experiment, followed by a visual task (WORDS). An impedance check followed while participants relaxed before proceeding.

Block 2 began with a second visual task (IMG), followed by an auditory task (MM), after which a second impedance check was performed, and a brief rest was provided. Block 3 concluded the session, beginning with an auditory task (SENT) and ending with a short (∼2-min) audio-visual task that required no intentional motor response.

It is important to acknowledge that starting the experiment with a meditation period may have influenced performance in the subsequent tasks. This potential effect remains unexamined in the present work, as its investigation lies outside the scope of this study and would require a separate, dedicated analysis in future research.

### 3.1 Preprocessing

**FIGURE 2:**
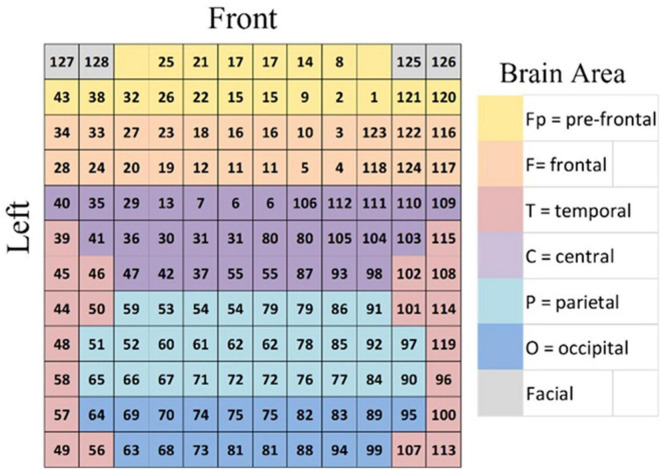
The 12 × 12 matrix (A) that includes the 128 EEG channels of the sensor net that is placed over the whole scalp. The matrix displays brain regions with different colors, as shown in the legend to the right of the matrix. The left and right hemispheres are represented in the matrix by two equal and symmetrical parts of two sub matrices of 12 × 6 each. Positions A_1,3_ and A_1,10_ are empty.

We recorded data with the use of the Net Station 5.0 at a sample rate of 1,000 Hz from 128 electrodes in a 128-vector. This vector was then transformed, after preprocessing, into a 12 × 12 matrix (A) representing each brain region of interest. We doubled a few electrodes to fill the matrix symmetrically. Positions A_1,3_ and A_1,10_ were left empty as a reference indicating the position of the pre-frontal cortex in the matrix (see Figure 2).

The present study makes no attempt to solve the EEG inverse problem, since addressing it lies far beyond the scope of our work. Our approach uses a simplified approximation in which subsets of scalp electrodes serve as proxies for underlying cortical activities. This mapping reflects scalp topology rather than true neural source localization, since electrode positions only approximate the brain areas beneath them and are unable to uniquely identify intracranial generators. Given the pilot nature of the study and the small sample size of 20 participants, these constraints admittedly represent an important limitation. Future, more comprehensive studies may incorporate source-modeling or anatomically informed forward models to refine spatial precision. Even with these constraints, the analyses reveal clear differences in scalp-level spatiotemporal dynamics across cognitive conditions, indicating robust patterns detectable at the sensor level.

We applied two methodologies described in detail in our recent publication (Davis et al., 2025). Two methodologies were presented: a Hilbert transform-based methodology and a Fourier transform methodology.

#### 3.1.1 Artifact rejection methods

All EEG recordings were preprocessed using a standardized artifact-reduction protocol applied consistently across studies (Davis and Kozma, 2018; Davis et al., 2023b; Davis et al., 2024b; Khoshnevis and Sankar, 2020). The procedure follows the general methodology introduced by Davis and Kozma (2018) and incorporates established practices in EEG signal conditioning and movement-artifact attenuation.

The raw signals were first detrended to remove slow drifts and low-frequency fluctuations associated with body motion and baseline instability. A 50 Hz notch filter was then applied to suppress power-line interference. To further isolate physiologically meaningful oscillatory activity, the data were subjected to band-pass filtering, retaining frequencies from 2 to 48 Hz. This range excludes very low-frequency drift while minimizing contamination from high-frequency muscular artifacts.

The combined application of impedance checks, detrending, notch filtering, and controlled band-pass filtering reduced poor quality electrode signal and artifacts arising from blinking, muscle contractions, and general movement. These steps yield a stable and reliable signal suitable for the extraction of entropy measures, skewness coefficients, and other measures and indices used in subsequent analyses.

### 3.2 Information-theoretical and other indices

In order to describe different brain states for the 20 participants associated to the different modalities measured, we apply a series of indices that were devised in previous studies (Davis et al., 2023b; Davis et al., 2020). These information measures allow for the investigation of potential meaning structures that have been associated to:

(a)    Phase Transitions from disorder to order and vice versa, in nonlinear dynamics, which describe the emergence and disintegration of large-scale functional brain structures at criticality (Kozma and Freeman, 2016, 2017) that are the foundation required to study the Cycle of Creation of Knowledge and Meaning necessary for intentional actions and values-based decision making, as treated by Davis et al. (2015a).
(b)    Superstructures, as shown by Telesca and Lovallo (2011) and Ji (2019), respectively.

**FIGURE 3:**
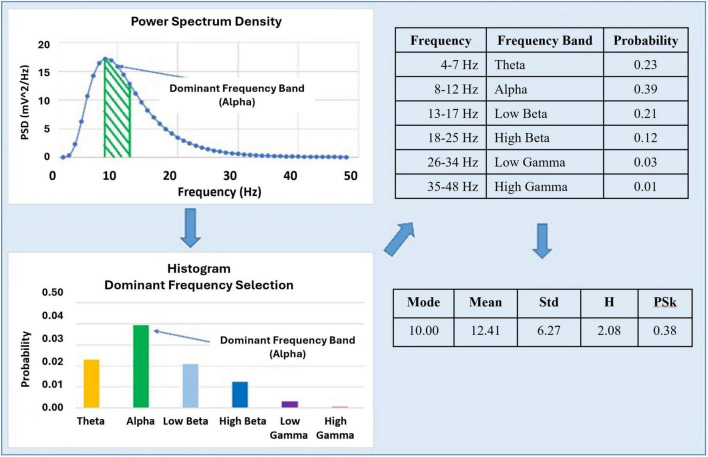
Illustrates how the H and PSk values are in general derived from the power spectrum, and particularly for PSk, from the dominant frequency band.

The two types of information measures derived from the normalized Power Spectrum are depicted in Figure 3.

The PSk coefficient is derived from the mean, standard deviation and mode (DF) empirical probability distribution, and the H index from the empirical probability distribution *per se*. TP is derived from the power spectrum.

When interpreting the PSk and H indices we invoke their definitions, as follows:

(a)    Entropy measure (H) as introduced by Shannon and Weaver (1971), giving the degree of randomness in an EEG signal.
(b)    PSk as a measure of structure described by the degree of asymmetry of a distribution, as derived by Pearson (1895) and discussed by Groeneveld and Meeden (1984). The version of PSk we use is the 1st order skewness coefficient, described and formulated in Weisstein (2022) based on the mean, standard deviation and mode.

From each electrode in the 12 × 12 matrix, we compute the temporal power spectrum (PSD_*t*_) over time windows of 500 ms, where the index “t” in PSD_*t*_ refers to a time window. From the PSD_*t*_ for each window we compute the Shannon entropy or diversity index (H), and the Pearson’s first skewness coefficient (PSk), the Total Power (TP) and the Dominant Frequency based (DF & DFs) indices. For the computation of H and PSk, we normalize the power spectrum to turn it into an empirical probability distribution. In previous studies, we have shown that H and PSk are good indices to classify different kinds of brain dynamics associated to different cognitive states (Davis et al., 2023b; Davis et al., 2020). However, in this study we introduce an approach that sets an overall conservative criterion to consider the results for the unequal variance *t*-tests of hypothesis, where we test for equal means μ1, μ2 between a pair of modalities (H0: μ1 = μ2) for these indices when taken together, resulting in overall Reject, Neutral or Accept, as will be shown in section 5.

Some timing corrections are applied to align recorded event times like “press key” with the recorded EEG data. The first correction is of eight ms for anti-alias filter, that accounts for the required time of the amplifier to do the conversion. The second correction is of 14 ms for the screen refresh rate, which amounts to a 22 ms shift to match the recorded event markers with the actual event time.

In the following section we show in detail the computation of H, PSk and TP (see Equations 1–3 respectively). Here DF (*Mode*_*PSD*t_) becomes relevant to the computation of PSk. The DFs is treated later in section 5.

### 3.3 Computation of the H, PSk, TP, DF based indices

In this section we introduce the reader to the details associated with the equations describing the computations to obtain H and PSk based on Total Power (TP) and Dominant Frequency Band.^2^

Discretizing PSD_*t*_ as follows:


$$P\,S\,D_{\text{t}}≐P\,W_{i}\,\left(F\,B_{i}\right)\,\forall i,f\,o\,r\,e\,a\,c\,h\,t\,i\,m\,e\,w\,i\,n\,d\,o\,w\,t$$

where PW_*i*_ corresponds to the power of frequency band “i” (*FB*_*i*_), we derive H as:


$$H=-\sum_{i=1}^{n}p_{i}*l\,o\,g_{2}\,\left(p_{i}\right)$$ (1)

$$w\,h\,e\,r\,e\;p_{i}=\frac{P\,W_{i}}{T\,P}$$

and TP is the total power computed as:


$$T\,P=\sum_{i=1}^{n}P\,W_{i}$$ (2)

The equation for the computation of PSk is:


$$P\,S\,k=\left|\frac{\left(M\,e\,a\,n_{P\,S\,D_{\text{t}}}-M\,o\,d\,e_{P\,S\,D_{\text{t}}}\right)}{S\,D_{P\,S\,D_{\text{t}}}}\right|$$ (3)

The *Mode*_*PSD*t_ represents the dominant frequency band and the SD_*PSD*t_ represents the standard deviation of the PSD_*t*_, where power is taken as a function of frequency and therefore frequency band, described here as PW_*i*_ (FB_*i*_).

When computing H, we use the probabilities p_*i*_ derived from the PSD_*t*_, where the number of a particular band is described by a fixed number “”” for all PSD_*t*_ for each participant and modality, as follows:


$$P\,S\,D\,t≐P\,W_{i}\,\left(F\,B_{i}\right)\;\forall \;i=4,5,6,\ldots ,48\,H\,z$$

In some situations we may need to adjust H to a new value H_*c*_, as described by Rajaram et al. (2017), since H_*c*_ may be useful when comparing brain dynamics for different brain areas in different bands, participants, and modalities that depend on unique and specific probability distributions. This is left for future studies and remains outside of the scope of the present work.

## 4 Experimental results

**FIGURE 4:**
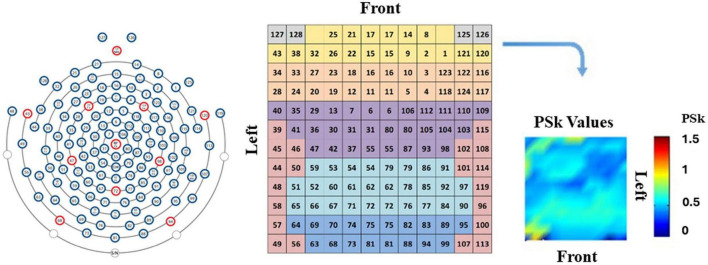
The EEG electrodes positions and numbers (left), the matrix representation of the EEG net (middle) and an example of a spatial 2D landscape displaying different brain areas (right), that could be derived from the mean values of H, PSk or any other measure of interest.

Following, we present a detailed analysis of brain dynamics data for all participants for the six modalities of interest. We have produced both qualitative and quantitative results that give sound and very useful information about the behavior of the H, PSk, TP, and DF based indices for the different participants in all modalities studied. We included data from every brain area represented by their associated electrodes, from where we computed the H, PSk, TP and DF based indices, as depicted in Figure 4, using PSk values as an example.

**FIGURE 5:**
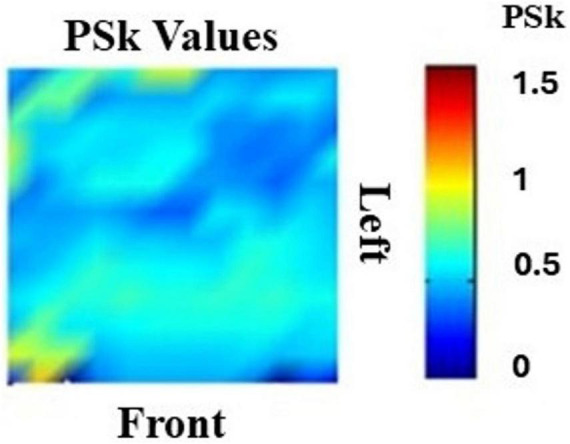
Shows a general 2D landscape by brain regions for the mean values of PSk.

We produce a set of brain dynamics landscape plots based on the mean values of H and PSk for each participant, for the six modalities, that give us a first impression about the brain dynamics differences and similarities between participants, modalities and brain regions. The landscapes described can be interpreted as the average of many pictures of the behavior of H or PSk in different windows of time “t.” These mean values are displayed for all the brain areas, where the back, front, left and right sides of the brain are clearly indicated, as shown in Figure 5. The color bar is indicative of the PSk mean values, for this example.

**FIGURE 6:**
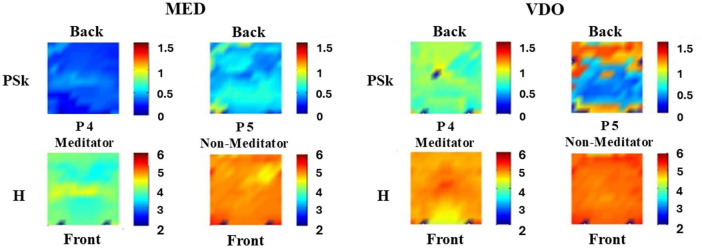
Shows the values for PSk (top row) and H (bottom row) for the different areas of the brain for Participant 4 (P 4) and 5 (P 5), Meditator and Non-Meditator respectively, in the modalities MED (left) and VDO (right).

We can clearly see in Figure 6 that the mean values for both H and PSk for both Participants (4 and 5) are significantly lower in the modality MED than in the modality VDO. Also, the H and PSk mean values for Participant 4, a meditator, are significantly lower than the mean values for Participant 5, a non-meditator, for both modalities.

We conduct a similar and exhaustive qualitative analysis of this kind, for all participants in both groups, meditators and non-meditators, for all six modalities, and observe significant and interesting differences between participants, modalities and groups.

**FIGURE 7:**
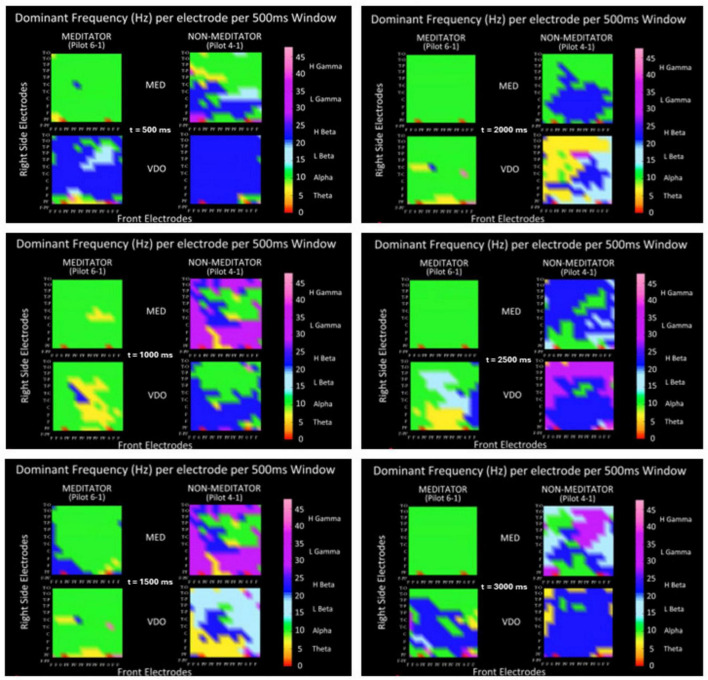
The values for the DF of brain dynamics as recorded in all areas of the scalp via a 12 × 12 matrix A, in six different moments, each a time window of 500 ms, showing the dominant frequency band as a particular color of the color bar for each moment or time window, for a meditator (left column of each picture) and a non-meditator (right column), in modalities MED (top row in each picture) and VDO (bottom row).

In another kind of qualitative analysis, we compute the dominant frequency band of the power spectrum in windows of 500 ms, in order to produce brain dynamic movies as means of encephalographic assessment, to visually discriminate between modalities. This proves to be very useful to train our perceptive apparatus, a natural neural network, that puts us in a similar position as that of a *sommelier* when discriminating between wines. Following, we show in Figure 7, several moments (frames) of such movies for modalities MED and VDO, for two participants, a meditator and a non-meditator.

**FIGURE 8:**
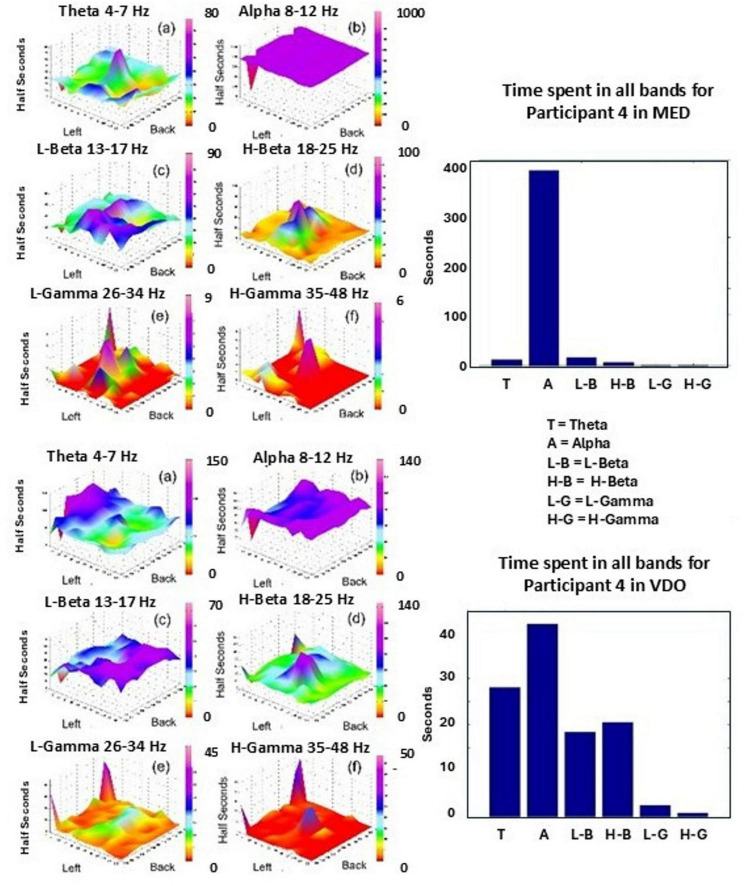
Shows total time (in seconds) that each band happens to be the dominant frequency band (see histograms, top and bottom). Surface plots **(a–f)** show total time (in half seconds) that each band happens to be the dominant frequency band for the different areas of the brain (left-right hemisphere, back-frontal areas), for Participant 4 in the modalities MED (top) and VDO (bottom).

The dominant frequency band of the power spectrum in windows of 500 ms also allows us to quantify the total time each frequency band happens to be the dominant frequency band, which serves as a measure of the dominance of each band in a particular modality. For example, in Figure 8, for Participant 4, we can appreciate that the Alpha band is the dominant frequency band for about 380 s of the total experimental time (420 s) for modality MED (top histogram). We also observe that Alpha is dominant for most brain regions of Participant 4, for most of the time, as clearly seen in Figure 8b, top right.

For the modality VDO, Alpha dominance still prevails, however, with significant activity in the Theta, L-Beta and H-Beta bands, as seen in Figure 8 (bottom histogram). The activity in Theta is predominant in the central prefrontal area, while Alpha and L-Beta are present predominantly in the occipital and parietal areas. H-Beta shows some activity, predominantly in the central area.

In a recent study (Davis et al., 2023b) we showed qualitatively that, generally speaking, when we compared the values for the *Meditator* vs. the *Non-Meditator* groups in both MED and VDO, the *Meditator* group members displayed lower mean values for H and PSk. Also, we noted that the overall variability displayed by H and PSk mean values was found to be greater for both groups (meditators and non-meditators) in the VDO modality than in MED, more prominent for the *Non-Meditator* group.

One interesting finding was the overall tendency for lower H and PSk mean values for a significant number of participants in the central frontal and central posterior region, particularly in modality MED. This is possibly due to the lack of visual stimuli (closed eyes), known to cause activity in the posterior part of the cortex, as shown in previous studies, where Alpha dominance is predominantly observed in the posterior parietal and occipital cortex, as measured via EEG (Barry et al., 2007; Berger, 1929; Miraglia et al., 2016).

Perhaps, the *Meditator* and *Non-Meditator* groups show Alpha dominance in the modality of MED due to participants meditating or relaxing with closed eyes. As found in a previous study, Alpha dominance is associated with low values of H and PSk (Davis et al., 2020).

Finally, we showed that apart from the Alpha dominance tendency, accompanied with low H and PSk mean values in modality MED for all participants, mainly in the occipital cortex, this tendency was also salient in the pre-frontal and frontal areas of the *Meditator* group members.

Now we are in the position to compare all modalities in order to evaluate them in terms of H, PSk, TP and DF based indices, individually and combined, for both the *Meditator and Non-Meditator* groups, via an appropriate quantitative statistical analysis.

## 5 Further results and analysis

In this section we show a detailed statistical analytical procedure to quantitatively compare groups and modalities for the H, PSk, TP and DFs, based on their respective mean values per electrode, per participant, per time window “t” of the Temporal Power Spectrum (PSD_*t*_).

**FIGURE 9:**
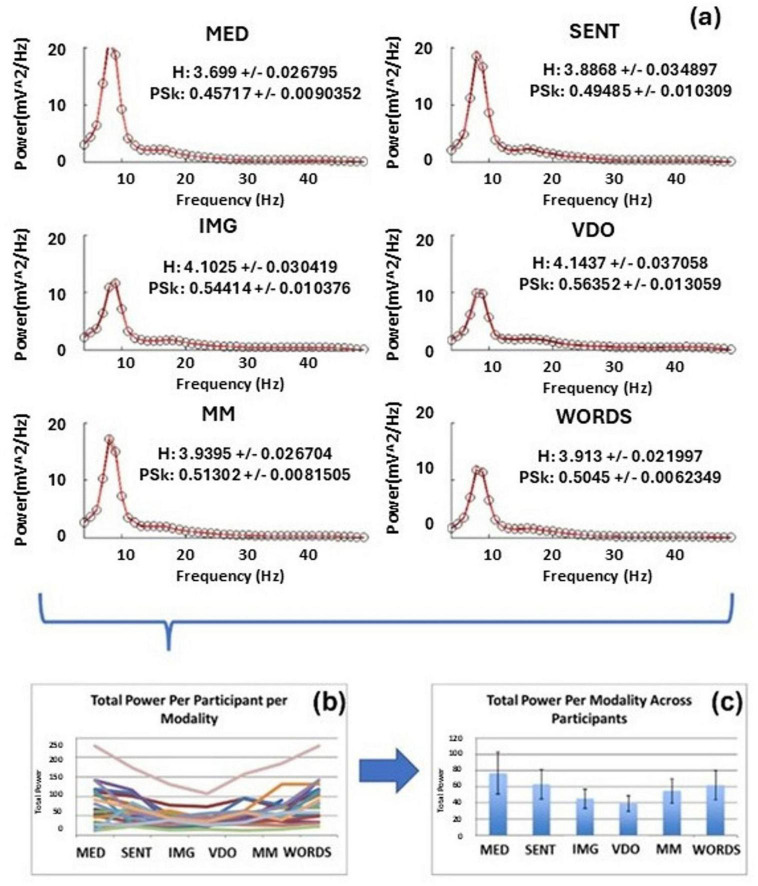
**(a)** the mean PSD over all time windows “t” for each modality for all participants (top), **(b)** TP per participant, per modality (bottom left) and **(c)** mean TP per modality with confidence intervals (bottom right).

We start by showing an example in Figure 9 of how to derive the Total Power (TP) per participant, per modality from their respective PSD_*t*_ (see equation 2). We also display the mean TP per modality with confidence intervals.

In order to compute the statistical parameters required for the analysis, based on H, PSk, TP and DFs, where DFs is the standard deviation of the mean of all dominant frequencies (see Equations 4, 5) across all electrodes and windows, we use the following formulas:


$${\bar{M\,o\,d\,e}}^{e}=\frac{\sum_{t=1}^{N\,W}M\,o\,d\,e_{P\,S\,D_{\text{t}}}^{e}}{N\,W}$$ (4)

where,


$$D\,F\,s=S\,T\,D\,\left({\bar{M\,o\,d\,e}}^{e}\right)\;\forall \;e=1,2,\ldots ,128$$ (5)

Following, we present some general definitions and the derivation of the formulas to be used.

Let us define $T\,P_{t,e}^{p,m}$ as the value of TP derived from the PSD_*t*_ in window *t*, for participant *p*, in modality *m*, for electrode *e*, where the number of windows (*NW*) in which we compute PSD_*t*_, is derived from the equation $N\,W\,\frac{L}{500}$, where *L* is the time length (ms) of a particular experiment for participant *p* in modality *m*, and where the length for each window *t* equals 500 ms.

We define the mean value of TP over all windows per electrode, per participant, per modality, as follows:


$${\bar{T\,P}}_{e}^{p,m}=\overset{N\,W}{\underset{t=1}{\sum }}\frac{T\,P_{t,e}^{p,m}}{N\,W}$$

Next, we define the mean value ${\bar{T\,P}}_{e}^{p,m}$ over all electrodes, per participant, per modality, as follows:


$${\bar{\bar{T\,P}}}^{p,m}=\overset{128}{\underset{e=1}{\sum }}\frac{{\bar{T\,P}}_{e}^{p,m}}{128}$$

Finally, we define the mean value of TP per group (g) per modality (m) as follows:


T⁢P¯gm=∑p=1n⁢p⁢gT⁢P¯¯p,mn⁢p⁢g ∀ g=1, 2

The reader should note that in the above formula, *npg* equals the *number of participants per group*, 1) for the *Meditator* group where g = 1, and 9 for the *Non-Meditator* group where g = 2. Similarly, we compute the standard deviation for the value of TP per group per modality, which we have labeled as T⁢P⁢sgm, from which we can derive the interval of confidence for T⁢P¯gm as follows:


T⁢P¯gm±t∝⁣=0.05,n⁢p⁢g*T⁢P⁢sgmn⁢p⁢g

The above formulas will apply to the computation of:


P⁢S⁢k¯ep,m,P⁢S⁢k¯¯p,m,P⁢S⁢k¯gm⁢a⁢n⁢d⁢P⁢S⁢k⁢sgm⁢H¯ep,m,H¯¯p,m,H¯gm⁢a⁢n⁢d⁢H⁢sgm,

D⁢F¯ep,m,D⁢F¯¯p,m,D⁢F¯gm⁢a⁢n⁢d⁢D⁢F⁢sgm 

**FIGURE 10:**
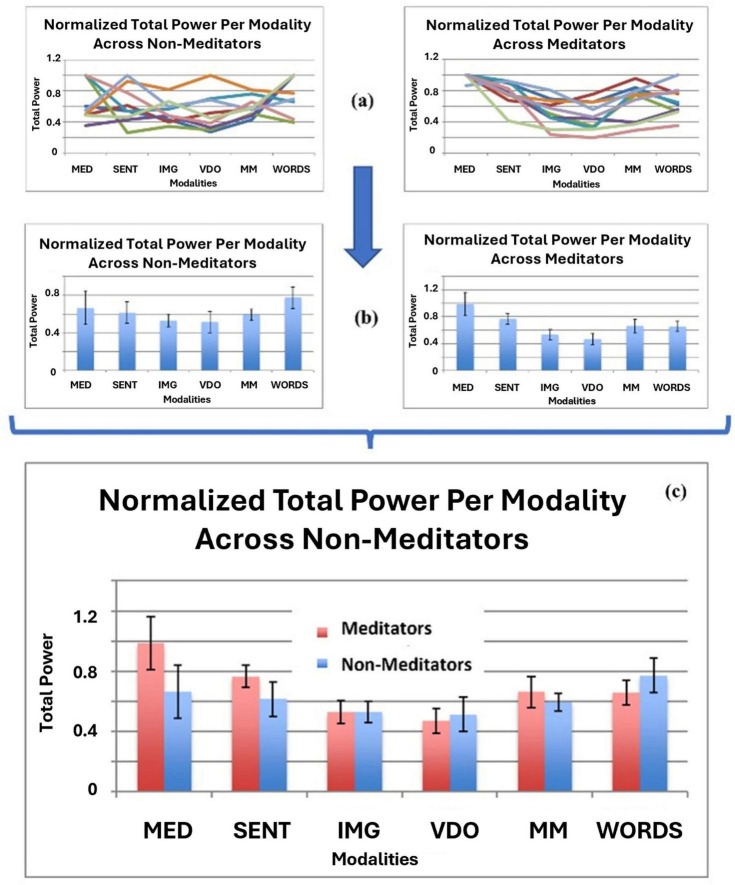
**(a)** Normalized TP per participant, per modality for the Non-Meditator group (top left) and the Meditator group (top right), **(b)** mean TP per modality with confidence intervals for the Non-Meditator group (middle left) and the Meditator group (middle right) and **(c)** a comparative bar chart with the normalized overall mean values for TP with confidence intervals, for the Meditator versus the Non-Meditator group in all modalities.

Throughout the paper, we use the labels H, PSk, TP and DF in a general sense, to denote the values of ${\bar{\bar{H}}}^{p,m},{\bar{\bar{P\,S\,k}}}^{p,m}$, ${\bar{\bar{T\,P}}}^{p,m}$, ${\bar{\bar{D\,F}}}^{p,m}$ respectively, when computing the landscapes, as shown in Figure 6, for example. However, H, PSk, TP and DF refer to, H¯gm,P⁢S⁢k¯gm⁢T⁢P¯gm⁢a⁢n⁢d⁢D⁢F¯gm respectively, when computing the intervals of confidence displayed in Tables 1, 2 and Figures 9, 10, for example. Here g = 1,2 for the Meditator and Non-Meditator group respectively, while *m* = 1, 2, 3, 4, 5, 6 for modalities MED, WORDS, IMG, MM, SENT, and VDO, respectively.

**TABLE 1:** Shows H overall mean values (${\bar{H}}_{1}^{1}$, ${\bar{H}}_{1}^{2}$, …, ${\bar{H}}_{1}^{6}$, and ${\bar{H}}_{2}^{1}$, ${\bar{H}}_{2}^{2}$, …${\bar{H}}_{2}^{6}$,) and their confidence intervals, for both groups in all modalities.

Group/modality	*Meditator*	*Non-Meditator*
MED	0.87 ± 0.03	0.92 ± 0.04
SENT	0.91 ± 0.04	0.96 ± 0.03
IMG	0.97 ± 0.01	0.98 ± 0.01
VDO	0.99 ± 0.01	0.99 ± 0.01
MM	0.96 ± 0.03	0.98 ± 0.02
WORDS	0.96 ± 0.02	0.97 ± 0.02

**TABLE 2:** Shows PSk overall mean values (P⁢S⁢k¯11,P⁢S⁢k¯12, …, P⁢S⁢k¯16 and P⁢S⁢k¯21,P⁢S⁢k¯22,P⁢S⁢k¯26) and their confidence intervals, for both groups in all modalities.

Group/modality	*Meditator*	*Non-Meditator*
MED	0.72 ± 0.05	0.76 ± 0.12
SENT	0.80 ± 0.04	0.86 ± 0.07
IMG	0.93 ± 0.02	0.96 ± 0.03
VDO	0.98 ± 0.02	0.99 ± 0.02
MM	0.91 ± 0.03	0.93 ± 0.06
WORDS	0.89 ± 0.03	0.93 ± 0.03

### 5.1 Meditators vs. Non-Meditators analysis

In this section, we conduct an analysis to compare the results of the two groups, *Meditator* and *Non-Meditator*.

In Figure 10 we observe that some modalities show significantly greater values than others for the mean TP. For example, modalities MED, SENT, MM and WORDS show greater mean TP values than modalities IMG and VDO. Also, modality MED shows significantly greater values for the *Meditator* group than all other modalities and the other group.

**FIGURE 11:**
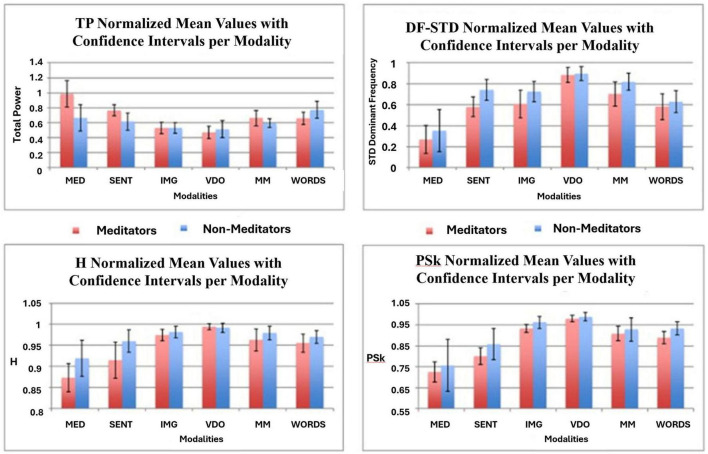
Displays the normalized mean values with confidence intervals for the Meditator group (red) and the Non-Meditator group (blue) for all six modalities in the TP (top left) index, the DFs (top right) index, the H (bottom left) index and the PSk (bottom right) index.

When comparing the H, PSk, TP, and DFs mean values for each modality in Figure 11, we observe that different modalities display statistically significant different mean values for all indices. Firstly we need to note that TP behaves in an opposite way to H, PSk, and DFs, therefore we have to restrict the comparison to H, PSk, and DFs. Here, H, PSk, and DFs show the smallest values in the modality MED, followed by the modality SENT, apart from the DFs index. On the other hand, modality VDO shows the largest mean values for all indices, together with the modality IMG, apart from the DFs index. The modalities MM and WORDS show similar mean values only for the H and PSk indices.

When we look at the behavior of TP, we observe, generally speaking, that the mean values display an opposite behavior to the other three indices, where for example MED shows the largest values, while IMG and VDO show the smallest. This is a very promising behavior, since individually, in general, the four indices are good at capturing the differences between modalities and groups. In Tables 1–4 we display the mean values for all indices in all modalities for both the *Meditator* and *Non-Meditator* group.

**TABLE 3:** Shows TP overall mean values (T⁢P¯11,T⁢P¯12,T⁢P¯16 and T⁢P¯21,T⁢P¯22,T⁢P¯26) and their confidence intervals, for both groups in all modalities.

Group/modality	*Meditator*	*Non-Meditator*
MED	0.99 ± 0.03	0.67 ± 0.17
SENT	0.77 ± 0.11	0.62 ± 0.11
IMG	0.53 ± 0.13	0.53 ± 0.07
VDO	0.47 ± 0.13	0.51 ± 0.11
MM	0.66 ± 0.16	0.59 ± 0.06
WORDS	0.66 ± 0.13	0.77 ± 0.12

**TABLE 4:** Shows DFs overall mean values (D⁢F⁢s¯11,D⁢F⁢s¯12,D⁢F⁢s¯16 and D⁢F⁢s¯21,D⁢F⁢s¯22,D⁢F⁢s¯26) and their confidence intervals, for both groups in all modalities.

Group/modality	*Meditator*	*Non-Meditator*
MED	0.27 ± 0.14	0.35 ± 0.20
SENT	0.58 ± 0.15	0.74 ± 0.10
IMG	0.61 ± 0.21	0.72 ± 0.10
VDO	0.88 ± 0.11	0.90 ± 0.07
MM	0.70 ± 0.18	0.82 ± 0.08
WORDS	0.58 ± 0.19	0.63 ± 0.11

It is important to note, however, that when we take a closer look at the mean values of H and TP in the MED and SENT modalities, we observe a statistically significant difference that may signal a distinct behavior in brain dynamics between the *Meditator* and *Non-Meditator* group for these two modalities.

**FIGURE 12:**
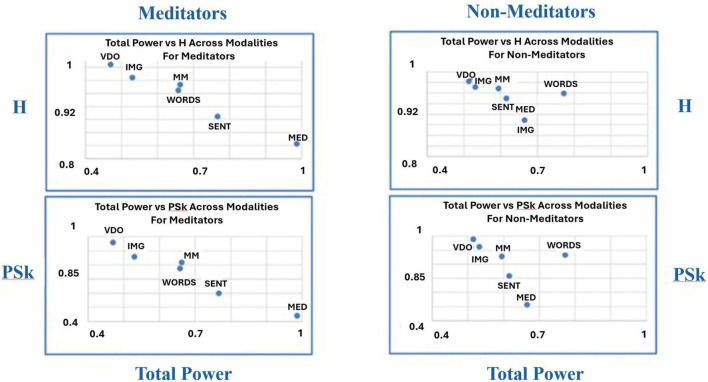
Shows the overall mean values for TP (x-axis) vs. PSk (y-axis, bottom) and H (y-axis, top) for both groups in all modalities.

**FIGURE 13:**
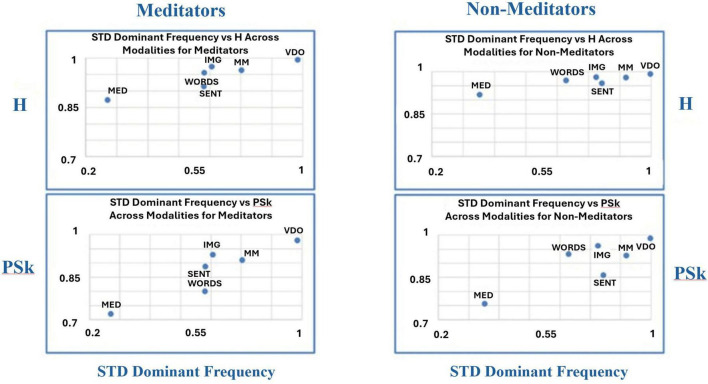
Shows the overall mean values for DFs (x-axis) vs. PSk (y-axis, bottom) and H (y-axis, top) for both groups in all modalities.

**FIGURE 14:**
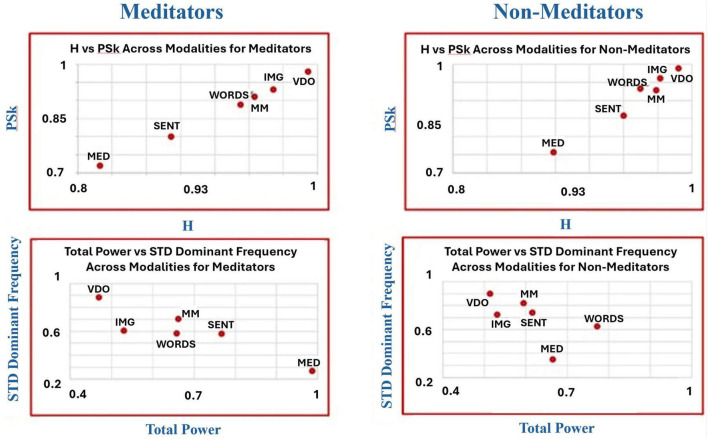
Shows the overall mean values for TP (x-axis, bottom) vs. DFs (y-axis, bottom) and H (y-axis, top) vs. PSk (y-axis, top) for both groups in all modalities.

To uncover potential additional differences and similarities between groups and modalities, we have produced another kind of analysis devised by Ji (2019) called “microstructure analysis,” where we plot a combination of a pair of indices in a two dimensional plot as means to discriminate between the behavior of the different modalities for the different groups, as shown in Figures 12–14.

In Figure 12 (top and bottom), we clearly observe that modality MED for the *Meditator* group shows the highest TP mean values, coupled with the smallest H and PSk mean values, followed in a linear fashion with a negative slope, by (a) modality SENT, (b) modalities MM and WORDS, (c) modality IMG, and (d) modality VDO. For the *Non-Meditator* group, we observe a more clustered behavior, where modality WORDS shows the highest TP mean values, with similar H and PSk mean values for all modalities, apart from the MED modality which shows the smallest H and PSk values. If we exclude the modality WORDS from the analysis, the tendency of the remaining modalities also shows a linear negative slope behavior, very similar for both H and PSk, in the following order: MED, SENT, MM, IMG, and VDO modalities. Note that the modalities VDO and IMG appear to be clustered together, as well as the MM and SENT modalities.

In Figure 13, we observe that modality MED for both the *Meditator* and *Non-Meditator* group shows the smallest DFs mean values coupled with the smallest H and PSk mean values, followed in a linear fashion with a positive slope, by (a) clustered modalities SENT, WORDS and IMG, (b) modality MM and (c) modality VDO showing the highest mean values for all DFs, H and PSk. Note that the clustered modalities SENT, WORDS and IMG show some variability between indices and groups.

Finally, in Figure 14 (top left and right) we observe that modality MED for both the *Meditator* and *Non-Meditator* group shows the smallest H mean values coupled with the smallest PSk mean values, followed in a linear fashion with a positive slope, by (a) modality SENT, (b) clustered modalities WORDS, MM, IMG and (c) modality VDO showing the highest mean values for H and PSk. Note that the clustered modalities WORDS, MM, IMG show some variability between indices and groups, and that for all modalities the mean values of PSk for the *Meditator* group are significantly smaller than the mean values for the *Non-Meditator* group.

When we look at Figure 14 (bottom left and right) we observe that modality VDO shows the highest DFs mean values coupled with the smallest TP mean values for both the *Meditator* and *Non-Meditator* group, followed by all other modalities in a linear fashion with a negative slope.

For the *Meditator* group the VDO modality is followed by: (a) modality IMG, (b) clustered modalities WORDS and MM, (c) modality SENT, and (d) modality MED with the smallest TP and DFs mean values. In contrast, for the *Non-Meditator* group the VDO modality is followed by: (a) modality IMG, (b) clustered modalities MM and SENT, (c) modality MED, and (d) modality WORDS with the highest TP mean values, while modality MED displays the smallest DFs mean values.

All this analysis indicates that we must use all indices combined, when attempting to discriminate between different brain states for different participants, in different modalities for both the *Meditator* and *Non-Meditator* groups.

Overall, we can state, based on our qualitative and quantitative analysis so far, that brain dynamics are unique for each participant and when represented via H, PSk, TP, and DFs mean values, certain patterns and tendencies can be observed for the different groups in the different modalities.

### 5.2 Comparison between modalities multiple indices analysis

Finally, in this section, we present a set of hypotheses tests to statistically evaluate the difference between modalities and groups for each index, and in so doing, draw a better understanding about the behavior of the different cognitive states associated with each modality, as represented by different brain behavior based on H, PSk, TP, DFs.

We compute the *p*-values for a level of significance α = 0.05, for various unequal variance *t*-tests of hypothesis, where we test for equal means μ1, μ2 between a pair of modalities (H0: μ1 = μ2), for each of the four indices H, PSk, TP and DFs, for both the *Meditator* and *Non-Meditator* group. This allows for a comparison between modalities for the *Meditator* (see values above the matrix diagonal, marked with dots, “.”) and *Non-Meditator* group (see values below the matrix diagonal, marked with dots, “.”), as shown in Tables 5–8.

**TABLE 5:** Displays a number in the set {0,1} that corresponds to a value of the set {accept, reject} respectively, as a result of an unequal variance *t*-test of hypothesis, where H0: μ1 = μ2, allowing for a comparison between a pair of modalities for the *Meditator* (above matrix diagonal) and *Non-Meditator* group (below matrix diagonal) based on the H index.

Vs.	MED	MM	WORDS	SENT	IMG	VDO
MED	.	1	1	0	1	1
MM	1	.	0	1	0	1
WORDS	1	0	.	0	0	1
SENT	0	0	0	.	1	1
IMG	1	0	0	0	.	1
VDO	1	0	1	1	0	.

**TABLE 6:** Displays a number in the set {0,1} that corresponds to a value of the set {accept, reject} respectively, as a result of an unequal variance *t*-test of hypothesis, where H0: μ1 = μ2, allowing for a comparison between a pair of modalities for the *Meditator* (above matrix diagonal) and *Non-Meditator* group (below matrix diagonal) based on the PSk index.

Vs.	MED	MM	WORDS	SENT	IMG	VDO
MED	.	1	1	1*	1	1
MM	1	.	0	1	0	1
WORDS	1	0	.	1	0	1
SENT	0	0	0	.	1	1
IMG	1	0	0	1	.	1
VDO	1	1	1	1	0	.

The symbol “*” indicates a value very close to α = 0.05 (1* means rejected, just).

**TABLE 7:** Displays a number in the set {0,1} that corresponds to a value of the set {accept, reject} respectively, as a result of an unequal variance *t*-test of hypothesis, where H0: μ1 = μ2, allowing for a comparison between a pair of modalities for the *Meditator* (above matrix diagonal) and *Non-Meditator* group (below matrix diagonal) based on the TP index.

Vs.	MED	MM	WORDS	SENT	IMG	VDO
MED	.	1	1	1	1	1
MM	0	.	0	0	0	0*
WORDS	0	0	.	0	0	1
SENT	0	0	0	.	1	1
IMG	0	0	1	0	.	0
VDO	0	0	1	0	0	.

The symbol “*” indicates a value very close to α = 0.05 (0* means accepted, just).

**TABLE 8:** Displays a number in the set {0,1} that corresponds to a value of the set {accept, reject} respectively, as a result of an unequal variance *t*-test of hypothesis, where H0: μ1 = μ2, allowing for a comparison between a pair of modalities for the *Meditator* (above matrix diagonal) and *Non-Meditator* group (below matrix diagonal) based on the DFs index.

Vs.	MED	MM	WORDS	SENT	IMG	VDO
MED	.	1	1	1	1	1
MM	1	.	0	0	0	0
WORDS	1	0	.	0	0	1
SENT	1	0	0	.	0	1
IMG	1	0	0	0	.	1
VDO	1	0	1	0	0	.

We assign a zero value if we accept H0, otherwise, if we reject H0, we assign the value of one. For example, in Table 5, when we compare the modality MED with modality VDO for the *Meditator* group, we reject H0, since we assign the value of one to the outcome of the test. In the same Table 5, when we compare the modalities SENT and WORDS for the *Non-Meditator* group, we accept H0, since the value of zero is assigned for the outcome of the test.

We condensed the results in Table 9, with an overall criterion for Rejects, Neutrals and Accepts, when all results for the unequal variance *t*-tests of hypothesis (H0: μ1 = μ2) are taken together from Tables 5–8. The overall criteria is as follows: (a) Reject when H0 is rejected for 3 or 4 indices (light purple), (b) Neutral or Ambiguous when H0 is rejected for only two indices (green), and (c) Accept when H0 is rejected for 0 or 1 indices (light peach). The values for the *Meditator* group are displayed above the matrix’s diagonal and the *Non-Meditator* group below.

**TABLE 9:** Shows overall Rejects, Neutrals and Accepts when all results for the unequal variance *t*-tests of hypothesis (H0: μ1 = μ2) are taken together from Tables 5–8: (a) Reject when H0 is rejected for 3 or 4 indices (light purple), (b) Neutral or Ambiguous when H0 is rejected for only 2 indices (green), and (c) Accept when H0 is rejected for 0 or 1 indices (light peach).

Vs.	MED	MM	WORDS	SENT	IMG	VDO
MED	∸	Reject	Reject	Reject	Reject	Reject
MM	Reject	∸	Accept	Neutral	Accept	Reject
WORDS	Reject	Accept	∸	Accept	Accept	Reject
SENT	Accept	Accept	Accept	∸	Reject	Reject
IMG	Reject	Accept	Accept	Accept	∸	Reject
VDO	Reject	Accept	Reject	Neutral	Accept	∸

The values for the *Meditator* group are displayed above the matrix’s diagonal and the *Non-Meditator* group below.

From Table 9 it is clear that overall, modality MED shows strong differences (Rejects) from the rest of the modalities for both the *Meditator* and *Non-Meditator* group, apart from the SENT modality for the *Non-Meditator* group (Accept). It is also clear that the VDO modality shows strong differences from the rest of the modalities, only for the *Meditator* group (Rejects). In contrast, the VDO modality, for the *Non-Meditator* group, seems to differ significantly for the modalities of MED and WORDS, yet showing similar behaviors in the modalities MM and IMG (Accepts), and a more neutral or ambiguous (Neutral) outcome for the SENT modality.

The modality MM for the Non-Meditator group shows no differences with the modalities WORDS, SENT, IMG, and VDO (Accepts). Modality SENT shows no differences with the modalities MED, MM, WORDS for Non-Meditators (Accepts) and weak or no differences with modalities VDO and IMG for Meditators (Neutral and Accept, respectively). Finally, modality IMG displays no differences with the modalities MM, WORDS for *Meditators* (Accepts) and MM, WORDS, and SENT for *Non-Meditators* (Accepts).

For the *Meditator* group the pair of modalities {WORDS, IMG} show no difference with modality MM (Accepts). Also, the pair of modalities {SENT, IMG} show no difference with modality WORDS for both *Meditators* and *Non-Meditators* (Accepts). Finally, the SENT modality ambiguity with modality MM for the *Meditator* group (Neutral), and modality VDO for the *Non-Meditator* group (Neutral).

In synthesis, above in Tables 10, 11, we group the results of H0 for all indices taken together in all pairs of modalities for the *Meditator* and *Non-Meditator* group, respectively, and we display in different colors the similarities between the two groups in terms of accepted and rejected results. This deepens our understanding of the differences between modalities when all indices are taken together, as shown in Table 9 by improving the quality of the analysis and our understanding of brain dynamics in different conditions or cognitive modalities.

**TABLE 10:** Shows the results of H0 between pairs of modalities for the *Meditator* group. We display in different colors the similarities between the two groups in terms of accepted and rejected result.

Modality	Accept	Reject	Neutral
MED		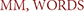 SENT, 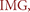 , 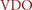	
MM	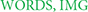	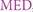 , VDO	SENT
WORDS	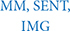	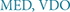	
SENT	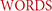	MED, IMG, VDO	MM
IMG	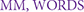	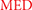 , SENT, VDO	
VDO		MED, MM, WORDS, SENT, IMG	

**TABLE 11:** Shows the results of H0 between pairs of modalities for the *Non-Meditator* group. We display in different colors the similarities between the two groups in terms of accepted and rejected result.

Modality	Accept	Reject	Neutral
MED	SENT	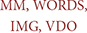	
MM	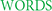 , SENT, 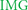 , VDO	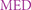	
WORDS	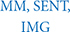	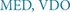	
SENT	MM, 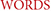 , SENT, IMG		VDO
IMG	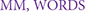 , SENT, VDO	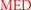	
VDO	MM, IMG	MED, WORDS	SENT

Our preliminary analysis includes an ANOVA test that revealed a significant effect of Modality on the measured index H, PSk, DFs and TP mean values from all participants. Since most modalities also passed multiple tests for normality for most indices via the Shapiro-Wilk, Anderson-Darling, D’Agostino K^2^, and Kolmogorov-Smirnov, where *p*-values were well above α = 0.05, we concluded that using parametric inference including multiple hypothesis comparison confidence-interval construction across modalities, aligns well with the distribution properties of the data. However, we also noticed that for indices DFs and TP some modalities failed the normality test. This indicates that non-parametric tests would be appropriate.

Given the exploratory nature of this pilot study, which included only 20 participants (11 Meditators and 9 Non-Meditators), the application of strict multiple-comparison corrections would likely provide limited additional interpretive value. Even so, we conducted pairwise comparisons with Bonferroni correction. These analyses showed that the MED condition produced significantly different results than modalities IMG, VDO, and MM, each with very large effect sizes for indices H and PSk. No other pairwise differences remained significant after correction.

These results indicate that the mean H and PSk values for all participants in the MED condition are reliably and substantially lower than those in the other modalities. Our analysis confirms this conclusion. The remaining modalities lack significant differences from one another.

Since, as mentioned before, the DFs and TP indices behave differently, we performed both parametric and non-parametric tests for all indices (see Supplementary material).

Normality diagnostics indicated acceptable distributional properties for PSk and H, while DFs and TP violated normality assumptions. Variance homogeneity was violated for TP only. Consequently, repeated-measures ANOVA and Friedman tests were applied. An inverse relation between TP and other indices was observed, for example, lower mean entropy values in MED corresponds to relatively higher mean TP values.

Across all four indices (PSk, H, DFs, TP), omnibus tests (ANOVA and Friedman) consistently revealed significant modality effects (all *p* < 0.001), with effect sizes ranging from moderate (*W* = 0.28 for TP) to strong (*W* = 0.62 for PSk).

Pairwise comparisons showed robust differences between MED vs. VDO across all indices and methods, confirming this as the most consistent modality contrast. MED vs. IMG and MED vs. MM also showed robust differences, detected across multiple indices and both parametric and non-parametric tests. VDO vs. WORDS was consistently, significantly different across indices, indicating strong separation between these modalities.

Other contrasts (e.g., MED vs. SENT, SENT vs. IMG/VDO/MM) were detected primarily by Wilcoxon tests, suggesting distributional sensitivity rather than mean differences. Several pairs (e.g., SENT vs. WORDS, MM vs. WORDS, IMG vs. MM) were consistently non-significant, supporting equality of means.

Overall, the consolidated evidence indicates that pairwise analyses demonstrated that MED yielded significantly different outcomes than IMG, VDO, and MM across all indices. No consistent differences were detected among IMG, VDO, MM, and WORDS. SENT showed an intermediate outcome.

We acknowledge the need for further benchmarking between statistical methods and for more rigorous statistical validation of these observations in future studies with a significantly larger sample size (for a comprehensive, detailed display of the above results (see Supplementary Table 34). It is important to note that this table presents many overlaps and differences between the results of parametric and non-parametric methods.

## 6 Discussion and future perspectives

### 6.1 The role of statistical indices to distinguish between meditators and non-Meditators

We have introduced the reader to a set of indices that when taken together, allow for a robust characterization of cognitive states, based on their associated brain dynamics, as captured by the power spectrum in 500 ms time windows (PSD_*t*_) in six modalities and two groups of participants (*Meditators* and *Non-Meditators*).

The indices we have used as classifiers are the Shannon Entropy Index (H), the Pearson’s 1st Skewness Coefficient (PSk), the Total Power of the power spectrum (TP), and the Standard Deviation of the mean dominant frequency band (DFs).

Our observations and results show that relaxed and meditative states result in Alpha dominance and relatively low values of H, PSk, and DFs with high values of TP.

We observe significant differences between the *Meditator* and the *Non-Meditator* group, more likely due to the effects of meditation in brain dynamics that may persist while engaged in other activities.

In general, it seems to us that meditative states are of low cognitive demand, followed by cognitive states associated with positive statement processing (SENT). The processing of scrambled words (WORDS), mental arithmetic operations (MM), and ambiguous images (IMG), impose greater cognitive demands than the more relaxed states already mentioned, followed by the most demanding sensory and cognitive states associated with watching a video with music and multiple displays of ambiguous images (VDO).

It is possible and likely that masterful meditators, people who practice relaxation or are naturally more relaxed, might be able to perform better or act more effectively and peacefully during the execution of demanding tasks and multi-sensory processing of environmental signals.

We also conjecture that this may possibly be due to the lasting psychophysiological effects derived from meditative practices (Davis et al., 2019b,2019a; Elbers and McCraty, 2020; McCraty et al., 2006), translating and continuing onto other areas of life.

This we can easily infer, for example, from the data associated with the modality of VDO when compared to the modality of MED, for both the *Meditator* and *Non-Meditator* group since both modalities are statistically different than the other modalities. Also, modality VDO behaves differently than all other modalities only for the *Meditator* group, while for the *Non-Meditator* group the outcomes are more variable when comparing modality VDO to the other modalities.

At this stage, it is still unclear how different modality SENT is from modality MED for both Meditators and Non-Meditators, since rejection or acceptance of the hypotheses seems to be a very tight decision, even though technically the outcomes have been statistically settled. The proximity between these two modalities can also be observed in their respective confidence intervals in Tables 1–4. It seems to us, based on our qualitative analysis and such proximity of these two modalities, that modality SENT shares similarities to relaxed or meditative states. A clearer distinction between these two modalities will require more data collection with new participants.

**FIGURE 15:**
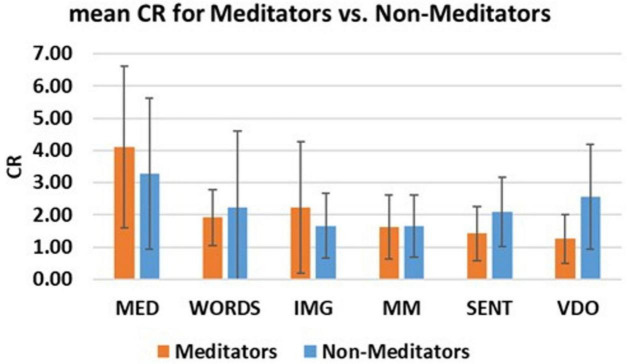
Shows the mean Coherence Ratio (CR) with error bars for each modality and for the Meditator (orange) and Non-Meditator (blue) group.

An additional index we also measured in this study, as shown in the following Figure 15, is the coherence ratio (CR), as an indicator of psychophysiological coherence derived from Heart Rate Variability (HRV) measures (Elbers and McCraty, 2020; McCraty et al., 2006).

We can clearly observe that the CR values for both groups are relatively larger for modality MED when compared to the other modalities, even in the presence of great variability. Again, this heart-rate-variability based index indicates the potential benefits of meditative and relaxed states when compared to more engaged states.

A deeper understanding of the relationship between brain-heart and respiration dynamics will require further studies with larger sample size and other psychophysiological measures, as treated by Kozma et al., (2022).

### 6.2 Future perspectives

In future work we intend to show how the brain participates in the creation of knowledge and meaning, as described by Davis et al. (2015b), and how this may be related to the neuro energetics of the brain in the modalities studied.

We propose that our methodology ought to be accompanied by first-person veridical reports describing the cognitive states under study, which may then be contrasted with responses generated by AI language models when presented with the same questions. This may prove to be very challenging when testing for human cognition and consciousness related to internal subjective states with the actual limitations of language that may limit veridical reports (Davis, 2020; Davis et al., 2024a; Diener et al., 1999; Metzinger, 2000; Ji and Davis, 2022). However, Lieberman (2025) shows some hope.

In the case of Large Language Models (LLM) this may just be impossible, since ChatGPT for example, has no subjective life. Byrne explains this tension in his abstract: *“I show that a conflict arises between active and passive consciousness when reading ChatGPT. While I actively know that there is no communicating subject, I still passively experience these signs as made by another. I argue that it is this conflict which lends ChatGPT its “magical” character. I conclude by showing how these observations can inform future regulation of AI models”* (Byrne, 2024).

Taken together with other studies, such as Davis (2009), Davis et al. (2015a), and Freeman (2008), we consider that this work has contributed to a better understanding of the relationship between brain dynamics and cognitive states when performing different tasks that are presumably associated with multiple different types of meanings, both behavioral and spiritual, when performing intentional actions and making decisions of the kind that all modalities in this study have provided.

### 6.3 Strengths and limitations of this study

Here we present the strengths and limitations of the present study, along with considerations for future research. The study benefits from a highly controlled and consistent EEG processing pipeline applied uniformly across all modalities, including rigorous artifact rejection and normalization procedures that minimize modality-specific noise and variance inflation. The use of high-density EEG (128 electrodes) and a repeated-measures design enhances internal validity by reducing between-subject variability, while variance homogeneity and the robustness of ANOVA assumptions indicate that observed modality effects reflect true differences rather than unequal dispersion or data quality. Several limitations should be acknowledged. Entropy values were averaged across time windows and electrodes, yielding a global, time-invariant measure of spectral complexity; this approach avoids pseudoreplication and stabilizes estimates, though it obscures temporal dynamics and spatially localized effects that may be modality-specific. Scalp channels exhibit volume conduction and spatial correlation, and averaging across electrodes further reduces effective dimensionality, limiting inference about regional neural processes. The number of stimuli differed across modalities (ranging from 10 to 28), resulting in unequal numbers of analysis windows and differences in the precision of modality-level entropy estimates, particularly for modalities with fewer stimuli; however, homogeneity-of-variance tests suggest this had no systematic bias on the results. The modest sample size of twenty participants, combined with substantial data compression into single modality-level summary values, constrains generalizability, sensitivity to inter-individual variability, and statistical power for multiple-comparison corrections; meaningful application of such corrections would likely require larger cohorts of 200–500 participants and may allow the use of advanced statistical classification and machine learning techniques. Finally, the study leaves the EEG inverse problem outside its scope and relies on scalp-level electrode groupings as practical approximations of underlying cortical regions, providing a topographic representation shaped by electrode placement; future larger-scale studies may incorporate source-modeling techniques to enhance spatial precision. Taken together, the findings reflect robust, global modality-dependent differences in EEG spectral entropy under strong experimental control, while offering clear directions for future research to expand sample sizes, apply advanced statistical methods, and improve spatial and temporal resolution.

## 7 Conclusion

In the same spirit that Carl Friedrich Gauss once uncovered elegant order beneath apparent complexity, this study seeks to reveal the hidden coherence within human cognition through EEG analysis, where coherent states are hypothesized to be associated with mental clarity, low stress relaxed states, *silent consciousness* à la Baars (2013) and *pure experience* à la James (1904). We examined six distinct modalities of mental and brain activity: Meditation, Scrambled Words, Ambiguous Images, Math Mind, Sentences, and Video Watching. We analyzed the data using Shannon Entropy (H), Pearson’s Skewness (PSk), Total Power (TP), and Dominant Frequency Spread (DFs) to reveal distinct neurophysiological signatures. These neurophysiological patterns suggest that cognition is far from merely a computational process, instead, it is an expression of deeper experiential and existential states. Just as Gauss reframed a problem through insight rather than brute calculation, our findings invite a re-examination of scientific inquiry itself, both as a pursuit of mechanistic regularities, and also, holistically, as a means of honoring the subtle interplay between structure and meaning. This interplay is vividly illuminated in meditative and relaxed states, like the ones manifested in modality MED, which consistently exhibits Alpha band dominance and lower entropy, reflected across multiple indices. The coherence ratio (CR), derived from heart rate variability, further supports the psychophysiological benefits of meditative states, suggesting a systemic resonance between brain, heart, and breath (Elbers and McCraty, 2020; McCraty et al., 2006), a triadic harmony that echoes the silent substratum of consciousness described by Baars (2013).

Baars’ Global Workspace Theory (GWT) (Baars, 1997) posits that consciousness emerges when information is globally broadcasted across specialized neural subsystems. In this framework, conscious access is an active integration, a dynamic spotlight illuminating salient content, instead of passive reflection. Yet Baars also gestures toward a subtler domain, “silent consciousness,” a state without reportable content, yet marked by awareness. This silent mode, often cultivated through contemplative repetition practices and near-threshold attending, may represent a workspace cleared of symbolic clutter, where coherence is presumably associated with pure experience à la James (1904), or pure transcendent and blissful consciousness, as in Zen practices, and itself becomes the content (Austin, 1999). Austin explains that “The EEG differs in one other respect besides frequency and amplitude during various forms and stages of meditation. This third characteristic is *coherence*,” (p. 89) associated with large scale synchronization at a significant number of electrodes. Our Alpha-dominant, low-entropy states may reflect this silent broadcasting, a neurophysiological signature of awareness without object.

These findings align with the broader framework of Freeman’s intentional neurodynamics, where cognition is seen as a field of embodied meaning (Davis and Gillett, 2023), instead of a computational artifact. At the mesoscopic level, wave packets emerge as carriers of intentional action, shaped by context, history, and ecological engagement (Gillett and Glannon, 2020). Baars’ *silent consciousness* complements this view, suggesting that the absence of content is far from implying the absence of cognition, rather its deepening, its retreat into the foundational substrate of awareness itself. Perhaps a form of neural background activity mediated by the default mode network (Brewer et al., 2011; Garrison et al., 2015; Simon and Engström, 2015). As Baars puts it, *“Silent consciousness may therefore correspond to increased theta-alpha power, spreading in cortex with minimal higher “content” frequencies, as has been reported during contemplative techniques”* (Baars, 2013).

This neurodynamic model stands in contrast to artificial neural networks and statistical models in silica, which lack the biological substrate and recursive intentionality that characterize human intelligence, cognition and consciousness. William Seager’s reflection on James and Bohm reinforces this distinction, proposing that consciousness is primary, woven into the fabric of reality as undivided wholeness, instead of emergent from complexity. In this view, rather than a machine, the human mind is a perspectival field, capable of generating meaning, through participation rather than simulation.

If artificial intelligence is to mimic cognition, it must grapple more than just informational entropy, and include at its core, the values and consciousness that give rise to meaning. The entropic balance of EEG signals may offer a window into coherence, yet only a species mature enough to honor life, liberty, and the pursuit of deep meaning, should attempt to design artificial “minds.” Taken together, our empirical results and philosophical reflections affirm that human intelligence arises through internal coherence, lived experience, and the sovereignty of the soul. The brain enacts rather than merely processing, and it resonates rather than computing (Gillett and Glannon, 2023). Meaning is revealed through intentional presence rather than extracted from data.

Future work will deepen this inquiry by examining how the brain participates in the creation of knowledge and meaning, and how neuroenergetic patterns reflect the spiritual and behavioral dimensions of intentional action. In honoring both the physics and metaphysics of cognition, we move closer to understanding the sacred jurisdiction of the human mind, a domain where intelligence is authentic, and never to be taken as artificial.

## 8 A final reflection: philosophical and normative considerations on consciousness and artificial intelligence

This discussion addresses empirical research: quantitative data, including neural indices, provide guidance for adjudicating questions related to individual subjective meaning, experiential interiority, or personal value structures. Human cognition exhibits profound variability, and even within a single individual, comparable tasks often generate distinct neural signatures. This variability, widely acknowledged in cognitive neuroscience (De Felice and Holland, 2018; Del Bene et al., 2025; Lange et al., 2022; Waschke et al., 2021), introduces inherent challenges when extending empirical findings into the domain of subjective experience. A deeper understanding of the human brain and consciousness must be achieved before engaging in the construction of artificial systems designed to emulate human-level intelligence or agency. These results delineate an epistemological boundary, providing a conceptual frame accompanying the empirical results (Journal of Consciousness Studies, 2024, 2025).

A normative perspective emerges as a central element of responsible scientific discourse. Scientific activity unfolds within a broader human context, and researchers carry an obligation to analyze data with rigor while addressing broader implications arising from their work. Moral reflections arise from empirical findings and conceptual inferences derived from these results.

In this concluding section, potential risks associated with emerging technologies engaging human cognitive capacities are outlined. The construction of meaning serves as an integrative synthesis, conveying a conceptual and ethical position grounded in the study’s findings, providing a context to the experimental measurement. Articulating the boundaries and limitations of specific experiments, alongside their ethical implications, contributes to transparent and conscientious scientific communication.

This reflection emphasizes human intelligence, neurodynamics, and the sovereignty of meaning. Here, sovereignty of meaning refers to autonomy, self-authorship, and self-governance. In the living brain, meaning emerges from the dynamic orchestration of neural fields, encompassing both quantum and classical mechanisms (Pribram, 2013; Schwartz et al., 2005). Freeman and Kozma’s neurodynamic framework demonstrates that cognition unfolds at the mesoscopic level, with transient, self-organizing patterns of electrical activity serving as carriers of intentional action. These neural patterns operate as expressions of embodied significance, shaped by history, context, and goal-directed activity, across multiple phase transitions (Kozma and Freeman, 2016, 2017).

Artificial neural networks (ANNs) and computational models operate through layered abstractions and optimization algorithms, whereas the human brain engages in metastable dynamics and resonant activity (Kelso and Engstrøm, 2006). Freeman’s intentional neurodynamics illustrate that perception and action form a recursive cycle, in which internal neural states reorganize continuously in response to ecological demands (Gillett and Glannon, 2023). These processes reflect active participation, extending beyond algorithmic simulation.

The material substrate of the brain, its wet, electrically active tissue, underlies the generation of meaning. Artificial systems lack such a biological substrate and wave-based neurodynamics. Their operational intelligence remains statistical, reflecting learned patterns in data without the first-person intentional arc characteristic of human experience (Merleau-Ponty, 2006; Dreyfus, 2002).

Seager’s essay, James, Bohm, and the Puzzle of Consciousness (Seager, 2025), highlights the convergence between William James’s radical empiricism and David Bohm’s implicate order, presenting consciousness as a manifestation of undivided wholeness rather than a computational artifact. James’s stream of consciousness (James, 1890, Chapter IX) and Bohm’s enfolded reality (Bohm, 1980, Chapter 3) indicates a non-local, non-linear intelligence that resisted algorithmic capture so far. Consciousness, subjective experience, and volition emerge as primary phenomena woven into reality.

The contrast between human and artificial intelligence becomes apparent. Artificial systems rely on external mappings, trained on data and optimized for performance. Human intelligence arises through internal coherence, shaped by lived experience, bodily resonance, and neural dynamics. Neurodynamics provide the biophysical basis, and philosophical frameworks illuminate the conceptual and ethical dimensions of cognition. Acknowledging this distinction emphasizes the importance of human-centered approaches and safeguards the integrity of subjective experience within research and applied technologies.

## Data Availability

The raw data supporting the conclusions of this article will be made available by the authors upon request. Requests to access these datasets should be directed to joshua.davis@auckland.ac.nz.

## Footnotes

1 The words modalities and conditions are used interchangeably throughout this paper.

2 In this study Dominant Frequency and Dominant Frequency Band are both described with the same label DF, which mostly refers to the dominant frequency band, as described in Figure 3.
