# Supplementary material for: A study of different cognitive states for meditators and non-meditators with the use of multiple classification indices derived from the PSD of EEG data and lessons learned about cognitive states and the nature of intelligence in minds and machines
Source: Front Syst Neurosci. 2026 Jan 23;19:1718733. doi: 10.3389/fnsys.2025.1718733 (PMC12876257; doi:10.3389/fnsys.2025.1718733)
Supplement: Supplementary file 1 [file Data_Sheet_1.pdf]

Supplementary Material

1. Normality Test Results for PSk

1.1 Shapiro-Wilk, D'Agostino, Anderson-Darling

Decision Rules

- Shapiro-Wilk: Reject normality if $p < 0.05$.
- D'Agostino K^2: Reject normality if $p < 0.05$.
- Anderson-Darling: Reject if statistic > critical value (here: 0.692 at 5%).

Supplementary Table 1. Shows the Shapiro-Wilk, D'Agostino Anderson-Darling Normality Test results.

Modality	Shapiro W	Shapiro p	D'Agostino K^2	D'Agostino p	Anderson Stat, 5% critical value = 0.692
**MED**	0.9592	0.5284	1.1764	0.5553	0.3245
**SENT**	0.9702	0.7590	0.9292	0.6284	0.2249
**IMG**	0.9566	0.4777	1.0818	0.5822	0.2925
**VDO**	0.9223	0.1097	6.9276	0.0313	0.3809
**MM**	0.9708	0.7712	1.0919	0.5793	0.1996
**WORDS**	0.9564	0.4754	1.6263	0.4435	0.2870

1.2 Homogeneity of Variance Tests

Test	Reject Homogeneity when
**Levene**	**p < 0.05**
**Brown–Forsythe**	**p < 0.05**
**Bartlett**	**p < 0.05**

Supplementary Table 2. Shows the Homogeneity of Variance Test results.

Test	Statistic	p-value
**Levene**	0.7309	0.6017
**Bartlett**	3.5937	0.6093
**Brown–Forsythe**	0.7309	0.6017

1.3 ANOVA Results

ANOVA tests the null hypothesis:

H_0: All group means are equal H_1: At least one group mean differs

Decision rule

- Reject H_0 if $p < \alpha$ (usually $\alpha = 0.05$).
- Fail to reject H_0 if $p \geq \alpha$.

1.3.1 Repeated-Measures ANOVA

Supplementary Table 3. Shows the Repeated-Measures ANOVA results.

Effect	F	Num DF	Den DF	Pr > F
**Modality**	32.5123	5	95	0.0000

1.3.2` Effect Sizes

How to interpret these Values:

Common interpretation guidelines (Cohen, 1988; Kirk, 1996).

Effect Size	Small	Medium	Large
**η^2**	~0.01	~0.06	~0.14
**ω^2**	~0.01	~0.06	~0.14

Supplementary Table 4. Shows the Effect Sizes and Values.

Effect Size	Value
η^2 (Eta Squared)	0.6312
ω^2 (Omega Squared)	0.6117

Both η^2 and ω^2 fall into the large effect size range.

1.4 Pairwise Comparisons (Bonferroni-Corrected)

Null hypothesis (H_0): The two Group means are equal.
Decision rule: reject H_0 when $p_{bonf} < \alpha$ (0.05). Fail to reject when $p_{bonf} \geq \alpha$.

Bonferroni correction multiplies the original p-value by the number of comparisons: $p_{\text{bonf}} = p \times m$, where m is the number of pairwise tests. This makes the test more conservative, reducing false positives. In this experiment m=15.

Supplementary Table 5. Shows the Pairwise Comparisons (Bonferroni-Corrected).

Comparison	t	p	p_{bonf}	d
**MED vs SENT**	-1.5386	0.1324	1.9865	-0.4865
**MED vs IMG**	-3.9131	0.0004	0.0063	-1.2374
**MED vs VDO**	-4.6547	0.0000	0.0007	-1.4720
**MED vs MM**	-3.2572	0.0025	0.0368	-1.0300
**MED vs WORDS**	-3.1232	0.0035	0.0531	-0.9877
**SENT vs IMG**	-2.5179	0.0163	0.2446	-0.7962
**SENT vs VDO**	-3.3467	0.0019	0.0284	-1.0583
**SENT vs MM**	-1.8514	0.0719	1.0790	-0.5855
**SENT vs WORDS**	-1.6901	0.0993	1.4890	-0.5345

**IMG vs VDO**	-0.9298	0.3583	5.3751	-0.2940
**IMG vs MM**	0.5778	0.5668	8.5026	0.1827
**IMG vs WORDS**	0.7884	0.4354	6.5307	0.2493
**VDO vs MM**	1.4432	0.1573	2.3591	0.4564
**VDO vs WORDS**	1.6657	0.1041	1.5612	0.5267
**MM vs WORDS**	0.1898	0.8505	12.7573	0.0600

1.5 Tukey HSD Post-Hoc Test

Null hypothesis (H_0): The two Group means are equal.
Decision rule: reject H_0 when reject = True (or the confidence interval does NOT include 0, or if p-adj < 0.05). You fail to reject when: reject = False (or the confidence interval includes 0, or if p-adj ≥ 0.05).

Supplementary Table 6. Shows the Turkey HSD Post-Hoc Test results.

Group1	Group2	meandiff	p-adj	Lower	Upper	Reject
**IMG**	**MED**	-0.1226	0.001	-0.2077	-0.0375	True
**IMG**	**MM**	-0.0157	0.9000	-0.1008	0.0694	False
**IMG**	**SENT**	-0.0708	0.1613	-0.1559	0.0143	False
**IMG**	**VDO**	0.0236	0.9000	-0.0615	0.1087	False
**IMG**	**WORDS**	-0.0211	0.9000	-0.1062	0.0640	False
**MED**	**MM**	0.1069	0.0054	0.0218	0.1920	True
**MED**	**SENT**	0.0518	0.4931	-0.0333	0.1369	False
**MED**	**VDO**	0.1461	0.0010	0.0610	0.2312	True
**MED**	**WORDS**	0.1015	0.0098	0.0164	0.1866	True

**MM**	**SENT**	-0.0551	0.4232	-0.1402	0.0300	False
**MM**	**VDO**	0.0392	0.7369	-0.0459	0.1243	False
**MM**	**WORDS**	-0.0054	0.9000	-0.0905	0.0797	False
**SENT**	**VDO**	0.0944	0.0206	0.0093	0.1795	True
**SENT**	**WORDS**	0.0497	0.5335	-0.0354	0.1348	False
**VDO**	**WORDS**	-0.0446	0.6320	-0.1297	0.0405	False

1.6 Friedman Test Decision Rule

Null hypothesis (H_0): All group distributions (medians) are equal across conditions.
Alternative hypothesis (H_1): At least one group distribution differs.

Decision Rule

Reject H_0 if the Friedman test p-value < α (commonly α = 0.05).
Fail to reject H_0 if the p-value ≥ α.

Effect Size (Kendall's W)

Friedman is often reported with **Kendall's W** as an effect size.
Interpretation of W:

W ≈ 0.1 - small effect.
W ≈ 0.3 - moderate effect.
W ≥ 0.5 - large effect.

Supplementary Table 7. Shows Friedman-Kendall Tests results.

Test	χ^2	df	p-value	Kendall's W
Friedman	62.4000	5	0.000000	0.6240

1.7 Wilcoxon Pairwise Comparisons

Null hypothesis (H_0): For each pair of modalities, the median difference between them = 0 (no difference). **Alternative hypothesis (H_1):** The median difference ≠ 0 (the two modalities differ).

Step-by-Step Decision Rule

Identify the corrected p-value (p_{bonf}) for each pair.

This is the Bonferroni-adjusted p-value reported in Table 5.

Compare p_{bonf} to α (usually 0.05).

If $p_{bonf} < 0.05$ - Reject H_0 - conclude the two modalities differ significantly.
If $p_{bonf} \geq 0.05$ - Fail to reject H_0 - conclude no statistically significant difference.

Effect size (r_{rb}) interpretation:

$|r_{rb}| \approx 0.1$ - small effect.
$|r_{rb}| \approx 0.3$ - moderate effect.
$|r_{rb}| \geq 0.5$ - large effect.

Negative values indicate the direction of difference (which modality tends to be lower).

Note: r_{rb} denotes the rank biserial correlation, an effect size recommended for Wilcoxon signed-rank tests. It quantifies the degree to which values from one condition tend to exceed values from another. Values of r_{rb} range from –1 to +1, where 0 indicates no systematic difference. Positive values indicate that the first condition tends to exhibit higher observed values, whereas negative values indicate lower observed values relative to the second condition. For example, $r_{rb} = 0.54$ reflects a large effect, indicating a strong tendency for the first condition to yield higher values than the second.

Values range from **–1 to +1**:

0: no difference.
Positive values: the first group tends to have higher scores.
Negative values: the first group tends to have lower scores.

Interpreting $r_{rb} = 0.5429 \approx 0.54$ - this indicates a large effect size.

Direction:

Since it's positive, it means the first group in the comparison tends to have higher values than the second group.

Supplementary Table 8. Wilcoxon Pairwise Comparisons.

Pair	W	p	p_{bonf}	r_{rb}
**MED vs SENT**	33.0	0.0056	0.0837	-0.6857
**MED vs IMG**	1.0	0.0000	0.0001	-0.9905
**MED vs VDO**	0.0	0.0000	0.0000	-1.0000
**MED vs MM**	5.0	0.0000	0.0003	-0.9524
**MED vs WORDS**	3.0	0.0000	0.0001	-0.9714
**SENT vs IMG**	2.0	0.0000	0.0001	-0.9810
**SENT vs VDO**	2.0	0.0000	0.0001	-0.9810
**SENT vs MM**	6.0	0.0000	0.0004	-0.9429
**SENT vs WORDS**	1.0	0.0000	0.0001	-0.9905
**IMG vs VDO**	15.0	0.0003	0.0039	-0.8571
**IMG vs MM**	65.0	0.1429	1.0000	0.3810
**IMG vs WORDS**	43.0	0.0192	0.2885	0.5905
**VDO vs MM**	31.0	0.0042	0.0633	0.7048
**VDO vs WORDS**	12.0	0.0001	0.0020	0.8857
**MM vs WORDS**	81.0	0.3884	1.0000	0.2286

There is a large difference between the two groups. The first group tends to score higher than the second group. Even if the corrected p-value was not significant, the effect size suggests the difference is meaningful.

2. Normality Test Results for H

2.1 Shapiro-Wilk, D'Agostino, Anderson-Darling

Decision Rules

- Shapiro-Wilk: Reject normality if $p < 0.05$.
- D'Agostino K^2: Reject normality if $p < 0.05$.
- Anderson-Darling: Reject if statistic > critical value (here: 0.692 at 5%).

Supplementary Table 9. Shows the Shapiro-Wilk, D'Agostino Anderson-Darling Normality Test results.

Modality	Shapiro W	Shapiro p	D'Agostino K^2	D'Agostino p	Anderson Stat, 5% critical value = 0.692
**MED**	0.9690	0.7330	0.8744	0.6458	0.1889
**SENT**	0.9696	0.7485	0.1194	0.9420	0.2371
**IMG**	0.9349	0.1917	7.1699	0.0277	0.3433
**VDO**	0.9072	0.0565	10.701	0.0047	0.5082
**MM**	0.9408	0.2484	2.6016	0.2723	0.4592
**WORDS**	0.9502	0.3708	4.1965	0.1226	0.4860

2.2 Homogeneity of Variance Tests

Test	Reject Homogeneity When
**Levene**	**$p < 0.05$**
**Brown–Forsythe**	**$p < 0.05$**
**Bartlett**	**$p < 0.05$**

Supplementary Table 10. Shows the Homogeneity of Variance test results.

Test	Statistic	p-value
**Levene**	2.1041	0.0699
**Bartlett**	8.9155	0.1125
**Brown–Forsythe**	2.1041	0.0699

2.3 ANOVA Results

ANOVA tests the null hypothesis:
H_0: All group means are equal H_1: At least one group mean differs.

Decision rule

- Reject H_0 if $p < \alpha$ (usually $\alpha = 0.05$).
- Fail to reject H_0 if $p \geq \alpha$.

2.3.1 Repeated-Measures ANOVA

Supplementary Table 11. Shows the Repeated-Measures ANOVA results.

Effect	F	Num DF	Den DF	Pr > F
**Modality**	25.6325	5.0000	95.0000	0.0000

2.3.2 Effect Sizes

How to interpret these Values:
Common interpretation guidelines (Cohen, 1988; Kirk, 1996).

Effect Size	Small	Medium	Large
**η^2**	~0.01	~0.06	~0.14
**ω^2**	~0.01	~0.06	~0.14

Supplementary Table 12. Shows the Effect Sizes and Values.

Effect Size	Value
η^2 (Eta Squared)	0.5743
ω^2 (Omega Squared)	0.5519

Both η^2 and ω^2 fall into the large effect size range.

2.4 Pairwise Comparisons (Bonferroni-Corrected)

Null hypothesis (H_0): The two Group means are equal.
Decision rule: reject H_0 when $p_{bonf} < \alpha$ (0.05). Fail to reject when $p_{bonf} \geq \alpha$.

Bonferroni correction multiplies the original p-value by the number of comparisons: $p_{\mathrm{bonf}} = p \times m$, where m is the number of pairwise tests. This makes the test more conservative, reducing false positives. In this experiment m=15.

Supplementary Table 13. Shows the Pairwise Comparisons (Bonferroni-Corrected).

Comparison	t	p	p_{bonf}	d
**MED vs SENT**	-1.5286	0.1347	2.0202	-0.4834
**MED vs IMG**	-3.7029	0.0008	0.0127	-1.1709
**MED vs VDO**	-4.3616	0.0001	0.0019	-1.3793
**MED vs MM**	-3.2223	0.0028	0.0414	-1.0190
**MED vs WORDS**	-2.8176	0.0079	0.1185	-0.8910
**SENT vs IMG**	-1.9712	0.0576	0.8633	-0.6233
**SENT vs VDO**	-2.6947	0.0110	0.1649	-0.8521
**SENT vs MM**	-1.5873	0.1212	1.8186	-0.5020
**SENT vs WORDS**	-1.1750	0.2477	3.7155	-0.3716

**IMG vs VDO**	-1.0217	0.3134	4.7015	-0.3231
**IMG vs MM**	0.3065	0.7610	11.4148	0.0969
**IMG vs WORDS**	0.8189	0.4182	6.2736	0.2590
**VDO vs MM**	1.2006	0.2375	3.5625	0.3797
**VDO vs WORDS**	1.6897	0.0995	1.4926	0.5343
**MM vs WORDS**	0.4650	0.6446	9.6693	0.1470

2.5 Tukey HSD Post-Hoc Test

Null hypothesis (H_0): The two Group means are equal.
Decision rule: reject H_0 when reject = True (or the confidence interval does NOT include 0, or if p-adj < 0.05). You fail to reject when: reject = False (or the confidence interval includes 0, or if p-adj ≥ 0.05).

Supplementary Table 14. Shows the Turkey HSD Post-Hoc Test results.

Group1	Group2	meandiff	p-adj	Lower	Upper	Reject
**IMG**	**MED**	-0.3394	0.0026	-0.5946	-0.0842	True
**IMG**	**MM**	-0.0226	0.9	-0.2778	0.2326	False
**IMG**	**SENT**	-0.1727	0.3717	-0.4279	0.0825	False
**IMG**	**VDO**	0.0689	0.9	-0.1863	0.3241	False
**IMG**	**WORDS**	-0.061	0.9	-0.3162	0.1942	False
**MED**	**MM**	0.3168	0.006	0.0616	0.572	True
**MED**	**SENT**	0.1667	0.4132	-0.0885	0.4219	False
**MED**	**VDO**	0.4083	0.001	0.1531	0.6635	True
**MED**	**WORDS**	0.2784	0.024	0.0232	0.5336	True

**MM**	**SENT**	-0.1501	0.527	-0.4053	0.1051	False
**MM**	**VDO**	0.0915	0.9	-0.1637	0.3467	False
**MM**	**WORDS**	-0.0384	0.9	-0.2936	0.2168	False
**SENT**	**VDO**	0.2416	0.0747	-0.0136	0.4968	False
**SENT**	**WORDS**	0.1117	0.7756	-0.1435	0.3669	False
**VDO**	**WORDS**	-0.1299	0.6579	-0.3851	0.1253	False

2.6 Friedman Test Decision Rule

Null hypothesis (H_0): All group distributions (medians) are equal across conditions.
Alternative hypothesis (H_1): At least one group distribution differs.

Decision Rule

Reject H_0 if the Friedman test p-value < α (commonly α = 0.05).
Fail to reject H_0 if the p-value ≥ α.

Effect Size (Kendall's W)

Friedman is often reported with **Kendall's W** as an effect size.
Interpretation of W:

W ≈ 0.1 - small effect.
W ≈ 0.3 - moderate effect.
W ≥ 0.5 - large effect.

Supplementary Table 15. Shows Friedman-Kendall Tests results.

Test	χ^2	df	p-value	Kendall's W
Friedman	55.3429	5	0.000000	0.5534

2.7 Wilcoxon Pairwise Comparisons

Null hypothesis (H_0): For each pair of modalities, the median difference between them = 0 (no difference). **Alternative hypothesis (H_1):** The median difference ≠ 0 (the two modalities differ).

Step-by-Step Decision Rule

Identify the corrected p-value (p_{bonf}) for each pair.

This is the Bonferroni-adjusted p-value reported in Table 13.

Compare p_{bonf} to α (usually 0.05).

If $p_{bonf} < 0.05$ - Reject H_0 - conclude the two modalities differ significantly.
If $p_{bonf} \geq 0.05$ - Fail to reject H_0 - conclude no statistically significant difference.

Effect size (r_{rb}) interpretation:

| r_{rb} | ≈ 0.1 - small effect.
| r_{rb} | ≈ 0.3 - moderate effect.
| r_{rb} | ≥ 0.5 - large effect.

Negative values indicate the direction of difference (which modality tends to be lower).
NOTE: r_{rb} stands for the rank-biserial correlation, which is the recommended effect size for Wilcoxon signed-rank tests. It quantifies the strength and direction of the difference between two paired groups.

Values range from **–1 to +1**:

0: no difference.
Positive values: the first group tends to have higher scores.
Negative values: the first group tends to have lower scores.

Interpreting $r_{rb} = 0.5429 \approx 0.54$ - this indicates a large effect size.

Direction:

Since it's positive, it means the first group in the comparison tends to have higher values than the second group.

Supplementary Table 16. Wilcoxon Pairwise Comparisons.

Pair	W	p	p_{bonf}	r_rb
**MED vs SENT**	19.0	0.0006	0.0088	-0.8190
**MED vs IMG**	2.0	0.0000	0.0001	-0.9810
**MED vs VDO**	0.0	0.0000	0.0000	-1.0000

**MED vs MM**	0.0	0.0000	0.0000	-1.0000
**MED vs WORDS**	0.0	0.0000	0.0000	-1.0000
**SENT vs IMG**	23.0	0.0012	0.0181	-0.7810
**SENT vs VDO**	11.0	0.0001	0.0016	-0.8952
**SENT vs MM**	19.0	0.0006	0.0088	-0.8190
**SENT vs WORDS**	41.0	0.0153	0.2297	-0.6095
**IMG vs VDO**	19.0	0.0006	0.0088	-0.8190
**IMG vs MM**	103.0	0.9563	1.0000	0.0190
**IMG vs WORDS**	55.0	0.0637	0.9558	0.4762
**VDO vs MM**	41.0	0.0153	0.2297	0.6095
**VDO vs WORDS**	15.0	0.0003	0.0039	0.8571
**MM vs WORDS**	62.0	0.1140	1.0000	0.4095

There is a large difference between the two groups. The first group tends to score higher than the second group. Even if the corrected p-value was not significant, the effect size suggests the difference is meaningful.

3 Normality Test Results for DFs

3.1 Shapiro-Wilk, D'Agostino, Anderson-Darling

Decision Rules

- Shapiro-Wilk: Reject normality if $p < 0.05$.
- D'Agostino K^2: Reject normality if $p < 0.05$.
- Anderson-Darling: Reject if statistic > critical value (here: 0.692 at 5%).

Supplementary Table 17. Shows the Shapiro-Wilk, D'Agostino Anderson-Darling Normality Test results.

Modality	Shapiro W	Shapiro p	D'Agostino K^2	D'Agostino p	Anderson Stat, 5% critical value = 0.692
**MED**	0.6866	2.69e-05	18.0914	0.000118	2.4440
**SENT**	0.9377	0.2165	1.9323	0.3805	0.3879
**IMG**	0.8503	0.00539	9.0125	0.0110	1.1643
**VDO**	0.9211	0.1039	4.3496	0.1136	0.5765
**MM**	0.9544	0.4391	1.2619	0.5321	0.3438
**WORDS**	0.8559	0.00671	13.5200	0.00116	0.8630

3.2 Homogeneity of Variance Tests

Test	Reject Homogeneity when
**Levene**	**p < 0.05**
**Brown–Forsythe**	**p < 0.05**
**Bartlett**	**p < 0.05**

Supplementary Table 18. Shows the Homogeneity of Variance Test results.

Test	Statistic	p-value
**Levene**	0.8343	0.5280
**Bartlett**	3.1905	0.6706
**Brown–Forsythe**	0.8343	0.5280

3.3 ANOVA Results

ANOVA tests the null hypothesis:
H_0: All group means are equal H_1: At least one group mean differs

Decision rule

- Reject H_0 if $p < \alpha$ (usually $\alpha = 0.05$).
- Fail to reject H_0 if $p \geq \alpha$.

3.3.1 Repeated-Measures ANOVA

Supplementary Table 19. Shows the Repeated-Measures ANOVA results.

Effect	F	Num DF	Den DF	Pr > F
**Modality**	13.6572	5	95	0.0000

3.3.2 Effect Sizes

How to interpret these Values:
Common interpretation guidelines (Cohen, 1988; Kirk, 1996).

Effect Size	Small	Medium	Large
η^2	~0.01	~0.06	~0.14
ω^2	~0.01	~0.06	~0.14

Supplementary Table 20. Shows the Effect Sizes and Values.

Effect Size	Value
η^2 (Eta Squared)	0.4182
ω^2 (Omega Squared)	0.3876

Both η^2 and ω^2 fall into the large effect size range.

3.4 Pairwise Comparisons (Bonferroni-Corrected)

Null hypothesis (H_0): The two Group means are equal.
Decision rule: reject H_0 when $p_{bonf} < \alpha$ (0.05). Fail to reject when $p_{bonf} \geq \alpha$.

Bonferroni correction multiplies the original p-value by the number of comparisons: $p_{\text{bonf}} = p \times m$, where m is the number of pairwise tests. This makes the test more conservative, reducing false positives. In this experiment m=15.

Supplementary Table 21. Shows the Pairwise Comparisons (Bonferroni-Corrected).

Comparison	t	p	p_{bonf}	d
**MED vs SENT**	-2.5387	0.0154	0.2305	-0.8028
**MED vs IMG**	-2.2160	0.0328	0.4915	-0.7008
**MED vs VDO**	-3.7549	0.0006	0.0095	-1.1874
**MED vs MM**	-3.1846	0.0029	0.0435	-1.0070
**MED vs WORDS**	-2.0117	0.0514	0.7714	-0.6362
**SENT vs IMG**	0.4504	0.6550	9.8250	0.1424
**SENT vs VDO**	-1.5300	0.1348	2.0219	-0.4838
**SENT vs MM**	-0.6209	0.5384	8.0760	-0.1963
**SENT vs WORDS**	0.4951	0.6234	9.3508	0.1566

**IMG vs VDO**	-1.9761	0.0563	0.8447	-0.6249
**IMG vs MM**	-1.1051	0.2762	4.1424	-0.3495
**IMG vs WORDS**	0.0735	0.9418	14.1274	0.0232
**VDO vs MM**	0.9928	0.3275	4.9122	0.3139
**VDO vs WORDS**	1.9540	0.0585	0.8776	0.6179
**MM vs WORDS**	1.1131	0.2726	4.0897	0.3520

3.5 Tukey HSD Post-Hoc Test

Null hypothesis (H_0): The two Group means are equal.
Decision rule: reject H_0 when reject = True (or the confidence interval does NOT include 0, or if p-adj < 0.05). You fail to reject when: reject = False (or the confidence interval includes 0, or if p-adj ≥ 0.05).

Supplementary Table 22. Shows the Tukey HSD Post-Hoc Test results.

Group1	Group2	meandiff	p-adj	Lower	Upper	Reject
**IMG**	**MED**	-1.0531	0.3649	-2.6002	0.4940	False
**IMG**	**MM**	0.5437	0.9000	-1.0034	2.0908	False
**IMG**	**SENT**	0.2220	0.9000	-1.3251	1.7691	False
**IMG**	**VDO**	1.1362	0.2801	-0.4109	2.6833	False
**IMG**	**WORDS**	-0.0364	0.9000	-1.5835	1.5107	False
**MED**	**MM**	1.5969	0.0389	0.0498	3.1440	True
**MED**	**SENT**	1.2751	0.1689	-0.2720	2.8222	False
**MED**	**VDO**	2.1894	0.0011	0.6423	3.7365	True
**MED**	**WORDS**	1.0167	0.4062	-0.5304	2.5638	False

**MM**	**SENT**	-0.3217	0.9000	-1.8688	1.2254	False
**MM**	**VDO**	0.5925	0.8661	-0.9546	2.1396	False
**MM**	**WORDS**	-0.5802	0.8793	-2.1273	0.9669	False
**SENT**	**VDO**	0.9142	0.5224	-0.6329	2.4613	False
**SENT**	**WORDS**	-0.2584	0.9000	-1.8055	1.2887	False
**VDO**	**WORDS**	-1.1727	0.2471	-2.7198	0.3744	False

3.6 Friedman Test Decision Rule

Null hypothesis (H_0): All group distributions (medians) are equal across conditions.
Alternative hypothesis (H_1): At least one group distribution differs.

Decision Rule

Reject H_0 if the Friedman test p-value < α (commonly α = 0.05).
Fail to reject H_0 if the p-value ≥ α.

Effect Size (Kendall's W)

Friedman is often reported with **Kendall's W** as an effect size.
Interpretation of W:

W ≈ 0.1 - small effect.
W ≈ 0.3 - moderate effect.
W ≥ 0.5 - large effect.

Supplementary Table 23. Shows Friedman-Kendall Tests results.

Test	χ^2	df	p-value	Kendall's W
Friedman	43.0	5	0.000001	0.43

3.7 Wilcoxon Pairwise Comparisons

Null hypothesis (H_0): For each pair of modalities, the median difference between them = 0 (no difference). **Alternative hypothesis (H_1):** The median difference ≠ 0 (the two modalities differ).

Step-by-Step Decision Rule

Identify the corrected p-value (p_{bonf}) for each pair.

This is the Bonferroni-adjusted p-value reported in Table 21.

Compare p_{bonf} to α (usually 0.05).

If $p_{bonf} < 0.05$ - Reject H_0 - conclude the two modalities differ significantly.
If $p_{bonf} \geq 0.05$ - Fail to reject H_0 - conclude no statistically significant difference.

Effect size (r_{rb}) interpretation:
$|r_{rb}| \approx 0.1$ - small effect.
$|r_{rb}| \approx 0.3$ - moderate effect.
$|r_{rb}| \geq 0.5$ - large effect.

Negative values indicate the direction of difference (which modality tends to be lower).
NOTE: r_{rb} stands for the rank-biserial correlation, which is the recommended effect size for Wilcoxon signed-rank tests. It quantifies the strength and direction of the difference between two paired groups.

Values range from **–1 to +1**:

0: no difference.
Positive values: the first group tends to have higher scores.
Negative values: the first group tends to have lower scores.

Interpreting $r_{rb} = 0.5429 \approx 0.54$ - this indicates a large effect size.

Direction:

Since it's positive, it means the first group in the comparison tends to have higher values than the second group.

Supplementary Table 24. Wilcoxon Pairwise Comparisons.

Pair	W	p	p_{bonf}	r_rb
**MED vs SENT**	4.0	0.0000	0.0002	-0.9619
**MED vs IMG**	20.0	0.0007	0.0106	-0.8095
**MED vs VDO**	0.0	0.0000	0.0000	-1.0000
**MED vs MM**	14.0	0.0002	0.0031	-0.8667

**MED vs WORDS**	6.0	0.0000	0.0004	-0.9429
**SENT vs IMG**	93.0	0.6742	1.0000	0.1143
**SENT vs VDO**	31.0	0.0042	0.0633	-0.7048
**SENT vs MM**	67.0	0.1650	1.0000	-0.3619
**SENT vs WORDS**	81.0	0.3884	1.0000	0.2286
**IMG vs VDO**	16.0	0.0003	0.0048	-0.8476
**IMG vs MM**	67.0	0.1650	1.0000	-0.3619
**IMG vs WORDS**	96.0	0.7562	1.0000	0.0857
**VDO vs MM**	51.0	0.0441	0.6608	0.5143
**VDO vs WORDS**	10.0	0.0001	0.0012	0.9048
**MM vs WORDS**	44.0	0.0215	0.3223	0.5810

There is a large difference between the two groups. The first group tends to score higher than the second group. Even if the corrected p-value was not significant, the effect size suggests the difference is meaningful.

4 Normality Test Results for TP

4.1 Shapiro-Wilk, D'Agostino, Anderson-Darling

Decision Rules

- Shapiro-Wilk: Reject normality if $p < 0.05$.
- D'Agostino K^2: Reject normality if $p < 0.05$.
- Anderson-Darling: Reject if statistic > critical value (here: 0.692 at 5%).

Supplementary Table 25. Shows the Shapiro-Wilk, D'Agostino Anderson-Darling Normality Test results.

Modality	Shapiro W	Shapiro p	D'Agostino K^2	D'Agostino p	Anderson Stat, 5% critical value = 0.692
**MED**	0.9122	0.0701	6.3075	0.0427	0.4631
**SENT**	0.8742	0.0140	9.5390	0.0085	0.7396
**IMG**	0.7974	0.0008	23.6671	0.000007	1.2135
**VDO**	0.7767	0.0004	22.2152	0.000015	1.4905
**MM**	0.8239	0.0020	18.8610	0.000080	1.1337
**WORDS**	0.8210	0.0018	18.4088	0.000101	1.1494

4.2 Homogeneity of Variance Tests

Test	Reject Homogeneity when
**Levene**	**$p < 0.05$**
**Brown–Forsythe**	**$p < 0.05$**
**Bartlett**	**$p < 0.05$**

Supplementary Table 26. Shows the Homogeneity of Variance Test results.

Test	Statistic	p-value
**Levene**	3.3677	0.0071
**Bartlett**	22.3948	0.0004
**Brown-Forsythe**	3.3677	0.0071

4.3 ANOVA Results

ANOVA tests the null hypothesis:
H_0: All group means are equal H_1: At least one group mean differs.

Decision rule

- Reject H_0 if $p < \alpha$ (usually $\alpha = 0.05$).
- Fail to reject H_0 if $p \geq \alpha$.

4.3.1 Repeated-Measures ANOVA

Supplementary Table 27. Shows the Repeated-Measures ANOVA results.

Effect	F	Num DF	Den DF	Pr > F
**Modality**	8.4651	5	95	0.0000

4.3.2 Effect Sizes

How to interpret these Values:
Common interpretation guidelines (Cohen, 1988; Kirk, 1996).

Effect Size	Small	Medium	Large
η^2	~0.01	~0.06	~0.14
ω^2	~0.01	~0.06	~0.14

Supplementary Table 28. Shows the Effect Sizes and Values.

Effect Size	Value
η^2 (Eta Squared)	0.308
ω^2 (Omega Squared)	0.272

Both η^2 and ω^2 fall into the large effect size range.

4.4 Pairwise Comparisons (Bonferroni-Corrected)

Null hypothesis (H_0): The two Group means are equal.
Decision rule: reject H_0 when $p_{bonf} < \alpha$ (0.05). Fail to reject when $p_{bonf} \geq \alpha$.

Bonferroni correction multiplies the original p-value by the number of comparisons: $p_{\text{bonf}} = p \times m$, where m is the number of pairwise tests. This makes the test more conservative, reducing false positives. In this experiment m=15.

Supplementary Table 29. Shows the Pairwise Comparisons (Bonferroni-Corrected).

Comparison	t	p	p_{bonf}	d
**MED vs SENT**	0.9240	0.3620	5.4301	0.2922
**MED vs IMG**	2.3256	0.0279	0.4189	0.7354
**MED vs VDO**	2.8445	0.0089	0.1339	0.8995
**MED vs MM**	1.5395	0.1341	2.0115	0.4868
**MED vs WORDS**	0.9679	0.3399	5.0989	0.3061
**SENT vs IMG**	1.7066	0.0974	1.4610	0.5397
**SENT vs VDO**	2.4019	0.0230	0.3446	0.7596
**SENT vs MM**	0.7123	0.4808	7.2125	0.2252
**SENT vs WORDS**	0.0521	0.9587	14.3804	0.0165

**IMG vs VDO**	0.8125	0.4218	6.3268	0.2569
**IMG vs MM**	-1.0733	0.2902	4.3533	-0.3394
**IMG vs WORDS**	-1.6513	0.1082	1.6236	-0.5222
**VDO vs MM**	-1.8545	0.0727	1.0910	-0.5864
**VDO vs WORDS**	-2.3470	0.0260	0.3897	-0.7422
**MM vs WORDS**	-0.6574	0.5151	7.7258	-0.2079

4.5 Tukey HSD Post-Hoc Test

Null hypothesis (H_0): The two Group means are equal.
Decision rule: reject H_0 when reject = True (or the confidence interval does NOT include 0, or if p-adj < 0.05). You fail to reject when: reject = False (or the confidence interval includes 0, or if p-adj ≥ 0.05).

Supplementary Table 30. Shows the Turkey HSD Post-Hoc Test results.

Group1	Group2	meandiff	p-adj	Lower	Upper	Reject
**MG**	**MED**	31.7505	0.0793	-2.0829	65.5838	False
**IMG**	**MM**	9.7361	0.9000	-24.0973	43.5695	False
**IMG**	**SENT**	17.7329	0.6328	-16.1005	51.5662	False
**IMG**	**VDO**	-5.9316	0.9000	-39.7649	27.9018	False
**IMG**	**WORDS**	17.0924	0.6641	-16.7410	50.9258	False
**MED**	**MM**	-22.0144	0.4178	-55.8477	11.8190	False
**MED**	**SENT**	-14.0176	0.8143	-47.8510	19.8157	False
**MED**	**VDO**	-37.6820	0.0197	-71.5154	-3.8487	True
**MED**	**WORDS**	-14.6581	0.7830	-48.4914	19.1753	False

**MM**	**SENT**	7.9968	0.9000	-25.8366	41.8301	False
**MM**	**VDO**	-15.6677	0.7337	-49.5010	18.1657	False
**MM**	**WORDS**	7.3563	0.9000	-26.4771	41.1897	False
**SENT**	**VDO**	-23.6644	0.3334	-57.4978	10.1689	False
**SENT**	**WORDS**	-0.6404	0.9000	-34.4738	33.1929	False
**VDO**	**WORDS**	23.0240	0.3653	-10.8094	56.8574	False

4.6 Friedman Test Decision Rule

Null hypothesis (H_0): All group distributions (medians) are equal across conditions.
Alternative hypothesis (H_1): At least one group distribution differs.

Decision Rule

Reject H_0 if the Friedman test p-value < α (commonly $\alpha = 0.05$).
Fail to reject H_0 if the p-value $\geq \alpha$.

Effect Size (Kendall's W)

Friedman is often reported with **Kendall's W** as an effect size.
Interpretation of W:

$W \approx 0.1$ - small effect.
$W \approx 0.3$ - moderate effect.
$W \geq 0.5$ - large effect.

Supplementary Table 31. Shows Friedman-Kendall Tests results.

**Test**	**χ^2**	**df**	**p-value**	**Kendall's W**
Friedman	28.37	5	0.000031	0.2837

4.7 Wilcoxon Pairwise Comparisons

Null hypothesis (H_0): For each pair of modalities, the median difference between them = 0 (no difference). **Alternative hypothesis (H_1):** The median difference ≠ 0 (the two modalities differ).

Step-by-Step Decision Rule

Identify the corrected p-value (p_{bonf}) for each pair.

This is the Bonferroni-adjusted p-value reported in Table 29.

Compare p_{bonf} to α (usually 0.05).

If $p_{bonf} < 0.05$ - Reject H_0 - conclude the two modalities differ significantly.
If $p_{bonf} \geq 0.05$ - Fail to reject H_0 - conclude no statistically significant difference.

Effect size (r_{rb}) interpretation:

$| r_{rb} | \approx 0.1$ - small effect.
$| r_{rb} | \approx 0.3$ - moderate effect.
$| r_{rb} | \geq 0.5$ - large effect.

Negative values indicate the direction of difference (which modality tends to be lower).
NOTE: $\mathbf{r_{rb}}$ stands for the rank-biserial correlation, which is the recommended effect size for Wilcoxon signed-rank tests. It quantifies the strength and direction of the difference between two paired groups.

Values range from **–1 to +1**:

0: no difference.
Positive values: the first group tends to have higher scores.
Negative values: the first group tends to have lower scores.

Interpreting $\mathbf{r_{rb}} = 0.5429 \approx 0.54$ - this indicates a large effect size.

Direction:

Since it's positive, it means the first group in the comparison tends to have higher values than the second group.

Supplementary Table 32. Wilcoxon Pairwise Comparisons.

Pair	W	p	p_{bonf}	r_rb
**MED vs SENT**	48.0	0.0328	0.4915	0.5429
**MED vs IMG**	29.0	0.0032	0.0473	0.7238
**MED vs VDO**	19.0	0.0006	0.0088	0.8190

**MED vs MM**	39.0	0.0121	0.1812	0.6286
**MED vs WORDS**	62.0	0.1140	1.0000	0.4095
**SENT vs IMG**	25.0	0.0017	0.0253	0.7619
**SENT vs VDO**	17.0	0.0004	0.0059	0.8381
**SENT vs MM**	61.0	0.1054	1.0000	0.4190
**SENT vs WORDS**	103.0	0.9563	1.0000	0.0190
**IMG vs VDO**	44.0	0.0215	0.3223	0.5810
**IMG vs MM**	29.0	0.0032	0.0473	-0.7238
**IMG vs WORDS**	14.0	0.0002	0.0031	-0.8667
**VDO vs MM**	13.0	0.0002	0.0025	-0.8762
**VDO vs WORDS**	14.0	0.0002	0.0031	-0.8667
**MM vs WORDS**	75.0	0.2774	1.0000	-0.2857

There is a large difference between the two groups. The first group tends to score higher than the second group. Even if the corrected p-value was not significant, the effect size suggests the difference is meaningful.

5 Omnibus Tests Across Indices

Supplementary Table 33. Shows Omnibus Test across Indices.

Index	ANOVA F (p)	η^2 / ω^2	Friedman χ^2 (p)	Kendall's W	Decision
**PSk**	F(5,95)=32.51, p<.001	η^2=0.63, ω^2=0.61 (large)	χ^2=62.40, p<.001	W=0.62 (strong)	Reject H_0
**H**	F(5,95)=25.63, p<.001	η^2=0.57, ω^2=0.55 (large)	χ^2=55.34, p<.001	W=0.55 (strong)	Reject H_0
**DFs**	F(5,95)=13.66, p<.001	η^2=0.42, ω^2=0.39 (large)	χ^2=43.00, p<.001	W=0.43 (moderate-strong)	Reject H_0
**TP**	F(5,95)=8.47, p<.001	η^2=0.31, ω^2=0.27 (large)	χ^2=28.37, p<.001	W=0.28 (moderate)	Reject H_0

All indices show significant modality effects with moderate to strong effect sizes.

5.1 Pairwise Comparisons (Consolidated)

PSk

- **Parametric (Bonferroni/Tukey):** MED vs IMG, MED vs VDO, MED vs MM, MED vs WORDS, SENT vs VDO significant.
- **Non-parametric (Wilcoxon):** MED differs from IMG, VDO, MM, WORDS; SENT differs from IMG, VDO, MM, WORDS; IMG vs VDO, VDO vs WORDS also significant.
- **Consensus:** MED consistently lower than others; SENT also differs strongly; VDO vs WORDS significant.

H

- **Parametric:** MED vs IMG, MED vs VDO, MED vs MM significant.
- **Non-parametric:** MED vs IMG, MED vs VDO, MED vs MM, MED vs WORDS; SENT vs IMG, SENT vs VDO, SENT vs MM; IMG vs VDO, VDO vs WORDS significant.
- **Consensus:** MED differs from IMG, VDO, MM robustly; SENT differs from IMG, VDO, MM; VDO vs WORDS significant.

DFs

- **Parametric:** MED vs VDO, MED vs MM significant.
- **Non-parametric:** MED vs SENT, MED vs IMG, MED vs VDO, MED vs MM, MED vs WORDS; IMG vs VDO, VDO vs WORDS significant.
- **Consensus:** MED differs from VDO and MM across both methods; Wilcoxon detects broader differences (distributional sensitivity).

TP

- **Parametric:** MED vs VDO significant; Tukey confirms MED vs VDO only.
- **Non-parametric:** MED vs IMG, MED vs VDO; SENT vs IMG, SENT vs VDO; IMG vs MM, IMG vs WORDS; VDO vs MM, VDO vs WORDS significant.
- **Consensus:** MED vs VDO robust across methods; additional Wilcoxon differences (MED vs IMG, SENT vs IMG/VDO, VDO vs WORDS).

5.2 Integrated Recommendation Table

Decision Rules:

REJECT = robust difference across tests.
EQUAL = robust equality across tests.
AMBIGUOUS = methods disagree (parametric vs non-parametric).
MIXED = indices disagree (different in one measure, equal in another).

Selection rules for Overall:

Rejects out of 4	Fails out of 4	Method disagreement	Overall
3 or 4	0 or 1	No	**Reject**
0 or 1	3 or 4	No	**Equal**
2	2	No	**Mixed**
If any methods disagree	If any methods disagree	Yes (parametric vs non-parametric conflict)	**Ambiguous**

Supplementary Table 34. Shows Integrated Recommendations.

Pair	PSk	H	DFs	TP	Overall Recommendation
**MED vs SENT**	Ambiguous	Ambiguous	Reject	Ambiguous	Ambiguous (Wilcoxon strong, parametric weaker)
**MED vs IMG**	Reject	Reject	Ambiguous	Reject	Different
**MED vs VDO**	Reject	Reject	Reject	Reject	Different (robust across all indices)
**MED vs MM**	Reject	Reject	Reject	Ambiguous	Different
**MED vs WORDS**	Reject	Reject	Ambiguous	Ambiguous	Ambiguous (Wilcoxon strong, parametric weaker)
**SENT vs IMG**	Reject	Ambiguous	Equal	Reject	Ambiguous
**SENT vs VDO**	Reject	Ambiguous	Equal	Reject	Ambiguous
**SENT vs MM**	Reject	Ambiguous	Equal	Reject	Ambiguous
**SENT vs WORDS**	Ambiguous	Equal	Equal	Equal	Equal
**IMG vs VDO**	Reject	Reject	Ambiguous	Ambiguous	Ambiguous
**IMG vs MM**	Equal	Equal	Equal	Reject	Ambiguous
**IMG vs WORDS**	Equal	Equal	Equal	Reject	Ambiguous
**VDO vs MM**	Ambiguous	Equal	Equal	Ambiguous	Ambiguous
**VDO vs WORDS**	Reject	Reject	Ambiguous	Reject	Different
**MM vs WORDS**	Equal	Equal	Equal	Equal	Equal
